# Alzheimer’s disease genetic risk and cognitive reserve in relationship to long-term cognitive trajectories among cognitively normal individuals

**DOI:** 10.1186/s13195-023-01206-9

**Published:** 2023-03-28

**Authors:** Corinne Pettigrew, Jurijs Nazarovs, Anja Soldan, Vikas Singh, Jiangxia Wang, Timothy Hohman, Logan Dumitrescu, Julia Libby, Brian Kunkle, Alden L. Gross, Sterling Johnson, Qiongshi Lu, Corinne Engelman, Colin L. Masters, Paul Maruff, Simon M. Laws, John C. Morris, Jason Hassenstab, Carlos Cruchaga, Susan M. Resnick, Melissa H. Kitner-Triolo, Yang An, Marilyn Albert

**Affiliations:** 1 Johns Hopkins University School of Medicine, 1600 McElderry St, Baltimore, MD 21205 USA; 2 University of Wisconsin-Madison School of Medicine and Public Health, 750 Highland Ave, Madison, WI 53726 USA; 3 Johns Hopkins Bloomberg School of Public Health, 615 N Wolfe St, Baltimore, MD 21205 USA; 4 Vanderbilt Memory and Alzheimer’s Center, Vanderbilt University Medical Center, 1207 17th Ave South, Nashville, TN 37212 USA; 5 John P. Hussman Institute for Human Genomics, University of Miami Miller School of Medicine, Miami, FL USA; 6 The Florey Institute, University of Melbourne, 30 Royal Parade, Parkville, VIC 3052 Australia; 7 Centre for Precision Health and Collaborative Genomics and Translation Group, Edith Cowan University, 270 Jundaloop Drive, Jundaloop, WA 6027 Australia; 8 Curtin Medical School, Curtin University, Kent Street, Bentley, WA 6102 Australia; 9 Washington University School of Medicine, 660 S Euclid Ave, St. Louis, MO 63110 USA; 10 National Institute on Aging Intramural Research Program, 251 Bayview Blvd, Baltimore, MD 21224 USA

**Keywords:** Alzheimer’s disease, Cognitive reserve, Genetics, Polygenic risk score, *APOE* genotype, Cognition, Cognitive decline

## Abstract

**Background:**

Both Alzheimer’s disease (AD) genetic risk factors and indices of cognitive reserve (CR) influence risk of cognitive decline, but it remains unclear whether they interact. This study examined whether a CR index score modifies the relationship between AD genetic risk factors and long-term cognitive trajectories in a large sample of individuals with normal cognition.

**Methods:**

Analyses used data from the Preclinical AD Consortium, including harmonized data from 5 longitudinal cohort studies. Participants were cognitively normal at baseline (*M* baseline age = 64 years, 59% female) and underwent 10 years of follow-up, on average. AD genetic risk was measured by (i) apolipoprotein-E (*APOE*) genetic status (*APOE-ε2* and *APOE-ε4* vs. *APOE-ε3*; *N* = 1819) and (ii) AD polygenic risk scores (AD-PRS; *N* = 1175). A CR index was calculated by combining years of education and literacy scores. Longitudinal cognitive performance was measured by harmonized factor scores for global cognition, episodic memory, and executive function.

**Results:**

In mixed-effects models, higher CR index scores were associated with better baseline cognitive performance for all cognitive outcomes. *APOE-ε4* genotype and AD-PRS that included the *APOE* region (AD-PRS_APOE_) were associated with declines in all cognitive domains, whereas AD-PRS that excluded the *APOE* region (AD-PRS_w/oAPOE_) was associated with declines in executive function and global cognition, but not memory. There were significant 3-way CR index score × *APOE-ε4* × time interactions for the global (*p* = 0.04, effect size = 0.16) and memory scores (*p* = 0.01, effect size = 0.22), indicating the negative effect of *APOE-ε4* genotype on global and episodic memory score change was attenuated among individuals with higher CR index scores. In contrast, levels of CR did not attenuate *APOE-ε4*-related declines in executive function or declines associated with higher AD-PRS. *APOE-ε2* genotype was unrelated to cognition.

**Conclusions:**

These results suggest that *APOE-ε4* and non-*APOE-ε4* AD polygenic risk are independently associated with global cognitive and executive function declines among individuals with normal cognition at baseline, but only *APOE-ε4* is associated with declines in episodic memory. Importantly, higher levels of CR may mitigate *APOE-ε4*-related declines in some cognitive domains. Future research is needed to address study limitations, including generalizability due to cohort demographic characteristics.

**Supplementary Information:**

The online version contains supplementary material available at 10.1186/s13195-023-01206-9.

## Background

In the coming decades, the prevalence and burden of Alzheimer’s disease (AD) and related dementias is projected to increase with the growth and aging of the population [1]. The lack of effective treatments for AD has led to an increased focus on potentially modifiable lifestyle factors that may mitigate dementia risk [2]. For example, variables reflecting lifetime cognitive experiences, such as more years of education, higher scores on literacy tests, and greater engagement in cognitively stimulating activities, are associated with better cognitive performance and a delayed onset of clinical symptoms of AD [3, 4]. These variables have been used as proxy measures of cognitive reserve (CR), a theoretical construct most recently defined as a property of the brain that allows for better-than-expected cognitive performance given age- and disease-related brain changes [5]. In contrast, several genetic risk factors increase the likelihood of cognitive decline and late onset AD dementia [6]. However, the extent to which CR proxy measures mitigate the relationship between genetic risk for AD and cognitive decline among individuals with normal cognition is not well understood.

The ε4 allele of the apolipoprotein-E gene (*APOE-ε4*) is a well-known risk factor for late onset AD dementia [7, 8], whereas the *APOE* ε2 allele (*APOE-ε2*) is associated with a reduced risk of AD dementia [9, 10]. Because other genetic factors additionally contribute to AD dementia risk, recent studies have examined polygenic risk scores for Alzheimer’s disease (AD-PRS), which combine the cumulative impact of multiple AD-associated genetic loci, as identified by genome-wide association studies (GWAS) (e.g., [6]). Like *APOE*, AD-PRS are also associated with increased risk of late onset AD dementia (e.g., [11–14]), though the magnitude of this risk tends to be smaller when removing the strong *APOE* effect from the score.

Studies examining the relationship of CR proxy measures and *APOE-ε4* carrier status to longitudinal cognitive decline have been most often conducted among individuals across the clinical spectrum (i.e., including combined groups of participants who are cognitively normal and have mild cognitive impairment (MCI) or those with normal cognition, MCI, and dementia). The results of these studies are mixed, with some finding that higher levels of CR attenuate *APOE-ε4*-related decline in global cognition [15–20], while others did not find such associations [21–25]. Moreover, studies among individuals with normal cognition at baseline are more limited. One study that included older adults (*M* age =75.9 years) reported that more years of education attenuated *APOE-ε4*-related declines in a memory composite score but not declines in language or visuospatial/reasoning composite scores [26]. However, other studies among largely middle-aged cohorts [27] or with smaller sample sizes [28] have reported no interaction between CR proxy variables and *APOE-ε4*. Additionally, to our knowledge, no prior studies have evaluated interactions between CR proxies and *APOE-ε2* in relationship to longitudinal cognitive decline, although this is an important question given prior findings suggesting greater protective effects of CR on risk of MCI symptom onset among *APOE-ε2* carriers relative to *APOE-ε2* noncarriers [29].

To our knowledge, only one prior study has examined whether CR proxy measures modify the impact of an AD-PRS on cognitive decline (for cross-sectional studies [30, 31]). In this study among older adults across the clinical spectrum, Shin et al. [32] found that one of several measures of cognitive activity engagement—reading books—attenuated the negative effect of an AD-PRS that included *APOE* on cognitive decline.

The present study addresses several issues that remain unresolved by prior literature. First, no prior studies have simultaneously examined whether CR proxy measures differentially interact with *APOE-ε4* genetic status, *APOE-ε2* genetic status, and AD-PRS in relationship to longitudinal cognitive trajectories. Second, few prior studies have examined these questions in individuals with normal cognition at baseline, yet this is important for identifying effective strategies for preventing or delaying future cognitive impairment. Third, most prior studies on this topic have used education as a proxy for CR. However, measures of literacy, either alone or in combination with other variables, may be more sensitive proxies for CR, given years of education does not reflect educational quality and remains largely stable after early adulthood [33]. Lastly, prior studies have often used measures of global cognition or individual test scores to measure longitudinal change. It is therefore unclear whether interactions between CR and genetic factors differ by cognitive domain. To address these gaps, this study examined whether a CR index score, combining both years of education and literacy test scores, modifies the relationship between AD genetic risk factors and longitudinal cognitive trajectories in a large sample of participants who were cognitively normal at baseline (*N* = 1819). We included both *APOE-ε4* and *APOE-ε2* genetic status and evaluated two versions of the AD-PRS—one without the *APOE* region and another with the *APOE* region. We also measured longitudinal cognitive performance with factor scores for global cognition, episodic memory, and executive function, to systematically explore the possibility of domain-specific effects.

## Methods

### Participants

Data for these analyses were derived from the Preclinical AD Consortium (PAC), which established large, harmonized datasets to examine questions of importance to the preclinical phase of AD that might be challenging to address with smaller sample sizes. The PAC datafiles combine data from 5 ongoing cohort studies examining the earliest phases of AD, including the Adult Children Study (ACS) [34], the Australian Imaging, Biomarker, and Lifestyle (AIBL) study [35], the Neuroimaging Substudy of the Baltimore Longitudinal Study of Aging (BLSA) [36], the Biomarkers of Cognitive Decline Among Normal Individuals (BIOCARD) study [37], and the Wisconsin Registry for Alzheimer’s Prevention (WRAP) [38]. Details about each cohort’s study design and exclusionary criteria have been described previously [35–39]. To be included in the PAC datafiles, each participant had to be cognitively normal at baseline and have at least one molecular biomarker (derived from cerebrospinal fluid (CSF) or positron emission tomography (PET)) collected while they were cognitively normal. The first visit at which a participant was cognitively normal and had a molecular biomarker collected was defined as the “PAC Baseline.” Molecular biomarkers were not considered in the present analyses.

Participants in all cohorts undergo longitudinal clinical and cognitive examinations, as well as medical, neurologic and psychiatric assessments and consensus diagnoses based on published criteria, e.g., the National Institute on Aging/Alzheimer’s Association criteria for MCI [40] and dementia [41]. Clinical and cognitive assessments are completed at regular intervals (e.g., every 12, 18, or 24 months) depending on each study’s design. Longitudinal neuroimaging (i.e., magnetic resonance imaging (MRI) and PET) and CSF are also collected at regular intervals (e.g., 24 months or 36 months). Clinical diagnoses were made without knowledge of biomarker measures. All participants provided written informed consent, and study protocols were approved by each site’s local institutional review board.

These analyses included participants who were cognitively normal at their first cognitive assessment and had both *APOE* genotypes and proxies for CR (i.e., years of education and scores on literacy tests: *N* = 1819; participants in AD-PRS analyses: *N* = 1175). For the purpose of this manuscript, “baseline” was defined as a participant’s first available cognitive test score (some of the sites provided data preceding the participant’s “PAC Baseline”; therefore, the first visit for a subset of participants in these analyses occurred before an individual’s “PAC Baseline”).

### Cognitive assessments

Comprehensive neuropsychological batteries administered to each cohort include standardized tests spanning the cognitive domains of episodic memory, executive function, language, visuospatial processing, attention, and processing speed. In order to combine raw cognitive data across all sites, longitudinal cognitive performance was measured with previously validated harmonized cognitive factor scores for (i) global cognition, including most available tests within a cohort; (ii) episodic memory, including verbal and visual episodic memory; and (iii) executive function, including tasks such as digit span, task switching, fluency, and set shifting (for details, see [42, 43]; see Supplementary Table 1, Additional File 1 for a list of tests included in each factor score).

The cognitive factor scores were generated from the raw cognitive data using item response theory (IRT) implemented in Mplus and followed an item banking approach [44]. This method allows for tests that are common to all cohorts, as well as tests unique to one or a few cohorts, to be combined into domain-specific factor scores for each participant at each visit, thereby utilizing all available data. Briefly, generation of factor scores entailed pre-statistical harmonization to identify unique and common items across datasets [45]; statistical co-calibration using confirmatory factor analysis (CFA) models separately for each study, for each cognitive domain; and examination of model fit and the quality of the link between each study, using simulation and testing for differential item functioning by study [46]. Following pre-statistical harmonization, an item banking approach [44] was used to serially estimate graded-IRT models separately within each dataset [47]. In each model, parameters (e.g., loadings and thresholds or intercepts) of new items were retained in the item bank for use in the subsequent models. In a final standardization step, all participants were pooled to estimate a CFA in which all item parameters were fixed to their previously estimated values, resulting in factor scores for each domain, integrated across the cohorts. To facilitate comparisons across factor scores, the harmonized values were z-scored based on each participant’s “PAC Baseline.”

### Cognitive reserve proxy index score

A harmonized CR index score was calculated by *z*-scoring and then averaging years of education and first available literacy test scores, consistent with a previously published method [48]. Literacy test scores reflected measures of verbal ability and reading, as often used in estimating crystallized intelligence. The literacy test scores were *z*-scored within cohort, given different assessments were administered across cohorts (ACS: Slosson Oral Reading Test [49]; AIBL: Wechsler Test of Adult Reading [50]; BIOCARD: National Adult Reading Test [51]; BLSA and WRAP: Wide Range Achievement Test 3 Letter and Word Reading [52]). The years of education variable was z-scored separately for cohorts within vs. outside the USA, given differences in educational systems.

### Genetic measures

DNA was extracted from whole blood in each study. GWAS data were generated from various genotyping arrays across studies with *APOE* genotyping performed separately using a targeted genotyping approach (see Supplementary Table 2, Additional File 1). *APOE* genetic status, provided by each site, was coded with separate dichotomous indicator variables for *APOE-ε2* (ε2/ε2 and ε2/3 = 1; otherwise 0), *APOE-ε3* (ε3/ε3 = 1, otherwise 0), and *APOE-ε4* (ε2/ε4, ε3/ε4 and ε4/ε4 = 1; otherwise 0). Individuals with ε2/ε4 alleles were included in the *APOE-ε4* group given their risk for AD pathology is similar to that of ε4 carriers, rather than ε2 carriers [53].

Raw GWAS data from each site were imputed by chip using a standard pipeline that included variant filtering for genotyping efficiency (95%), minor allele frequency (> 1%), and Hardy-Weinberg equilibrium (*p* > 1 × 10^−6^). Samples were removed for low call rate (< 99%) or for a mismatch between reported and genetically confirmed sex. Given the racial and ethnic makeup of the included studies, all GWAS analyses were also restricted to those of European ancestry that was confirmed using population principal component (PC) analysis. Individuals who did not self-report as non-Hispanic White or were more than 5 SDs away from the 1000 Genomes EU reference population based on PC analysis were removed. Imputation was performed on the TOPMed Imputation Server (version 1.6.0, https://imputation.biodatacatalyst.nhlbi.nih), and variants were filtered post-imputation to include common (> 1%) biallelic single nucleotide polymorphisms (SNPs) with a high imputation quality (*R* ^2^ > 0.8) and within expected Hardy-Weinberg Equilibrium (*p* > 1 × 10^−6^). Importantly, for the purpose of the AD-PRS analysis, we restricted all GWAS datasets to overlapping variants leaving a total of 6,739,456 common variants available in all five datasets for analysis.

AD-PRS were generated using imputed GWAS data, leveraging the summary statistics provided by Kunkle et al. [6] that were regenerated for us removing PAC participants who were included in the original GWAS analysis (*n* = 93,220). Original summary statistics are available at https://www.niagads.org/. Prior to generating the AD-PRS, linkage disequilibrium (LD) clumping was performed in PLINK (version 1.9; [54]) using a significance threshold for index SNPs of *p* = 0.01, LD clumping threshold of 0.5, and a window width of 200 kb. This threshold was based on a previous publication applying and evaluating multiple thresholds when generating an AD-PRS [12]. In order to generate scores on the same scale, variants were restricted to those common across all five datasets as outlined above. Following clumping and pruning performed in the largest genomic dataset (ACS), weights from 13,172 variants available in all datasets were used for the AD-PRS calculation and 12,948 variants available in all datasets were used for AD-PRS without the *APOE* region. AD-PRS generation was performed with PLINK using the method published previously [12], and scores were generated with and without the *APOE* region (i.e., 1 MB upstream and downstream of the *APOE* gene). For ease of reporting, the AD-PRS with and without *APOE* are abbreviated AD-PRS_APOE_ and AD-PRS_w/oAPOE_, respectively.

The two AD-PRS were transformed into *z*-scores to simplify interpretation, using the mean and standard deviation across all five datasets. To examine the impact of relatedness on the outcomes, two sets of AD-PRS were generated: one with all participants included in the measure and the other with related individuals excluded.

### Availability of data and materials

The plan is to archive the PAC datafiles at the National Archive of Computerized Data on Aging (NACDA). Investigators interested in accessing the data should contact the PAC Coordinating Center at Johns Hopkins University for details.

### Statistical analyses

Pearson correlations were used to evaluate the relationship between years of education and literacy test scores, separately for each cohort. Linear regressions were used to examine the relationship between AD genetic risk factors and the CR index score.

Linear mixed-effects models including random intercepts and slopes were used to examine the relationship of AD genetic risk factors and CR index score to cognitive trajectories, with separate models run for each cognitive factor score as the outcome. All models included the following predictors: baseline age, sex, terms for genetic status (i.e., *APOE* or AD-PRS), the CR index score, indicators for site (to control for site differences), time, and the interaction (i.e., cross-product) of each predictor with time, including time^2^ for evaluating non-linear (quadratic) trajectories. To examine whether the CR index score modifies the relationship between AD genetic risk and longitudinal cognitive trajectories, the models also included the 2-way CR index score × genetic status and 3-way CR index score × genetic status × time interactions. If the 3-way interaction was not significant, reduced models excluding this term were estimated. Baseline age and the CR index score were standardized (i.e., z-scored) across all cohorts before model fitting, and time was modeled in the unit of years (since baseline). All available follow-up was included.

Two sets of models were run. In the first set, “genetic status” reflected *APOE* genotype, as measured by indicators for *APOE-ε4* and *APOE-ε2* (with *APOE-ε3* as the reference group). In the second set, “genetic status” reflected the AD-PRS; these models were first run for AD-PRS_w/oAPOE_ then for AD-PRS_APOE_. Two sets of sensitivity analyses were run on the primary models. The first evaluated the relationship of the CR index score and AD genetic risk to longitudinal cognitive trajectories when individuals who progressed from normal cognition to MCI or dementia were excluded from the analyses. The second evaluated whether years of education and literacy scores made unique contributions to cognitive performance. Two additional sets of sensitivity analyses were run for the AD-PRS. The first evaluated whether the pattern of results remained unchanged when the *n* = 64 related individuals were excluded from the models, and within this subgroup, whether the patterns of results remained unchanged when the first five population PCs were included as covariates (for ensuring results were not driven by any unmeasured population stratification due to genetic ancestry). The second evaluated whether the relationship between the AD-PRS_w/oAPOE_ and cognitive trajectories remained the same when terms for *APOE-ε4* genetic status (i.e., *APOE-ε4* and *APOE-ε4* × time) were included as additional model predictors.

Effect sizes were calculated from a Cohen’s d derived from the linear mixed-effects models. To standardize each independent variable’s effect on the level or change in the cognitive outcome, effect sizes were calculated based on the standard deviation (SD) for the random intercept or SD for the random slope, respectively. SDs for random effects came from a reduced linear mixed-effects model that included only intercept and time as fixed effects and random effects.

Estimates (95% confidence intervals), *p*-values (with a significance level of *p* < 0.05), and effect sizes are reported. The mixed-effects models were run using the “lmer” function from “lmerTest” package in R (version 3.6.3) and Stata (version 17.0).

## Results

**Fig. 1:**
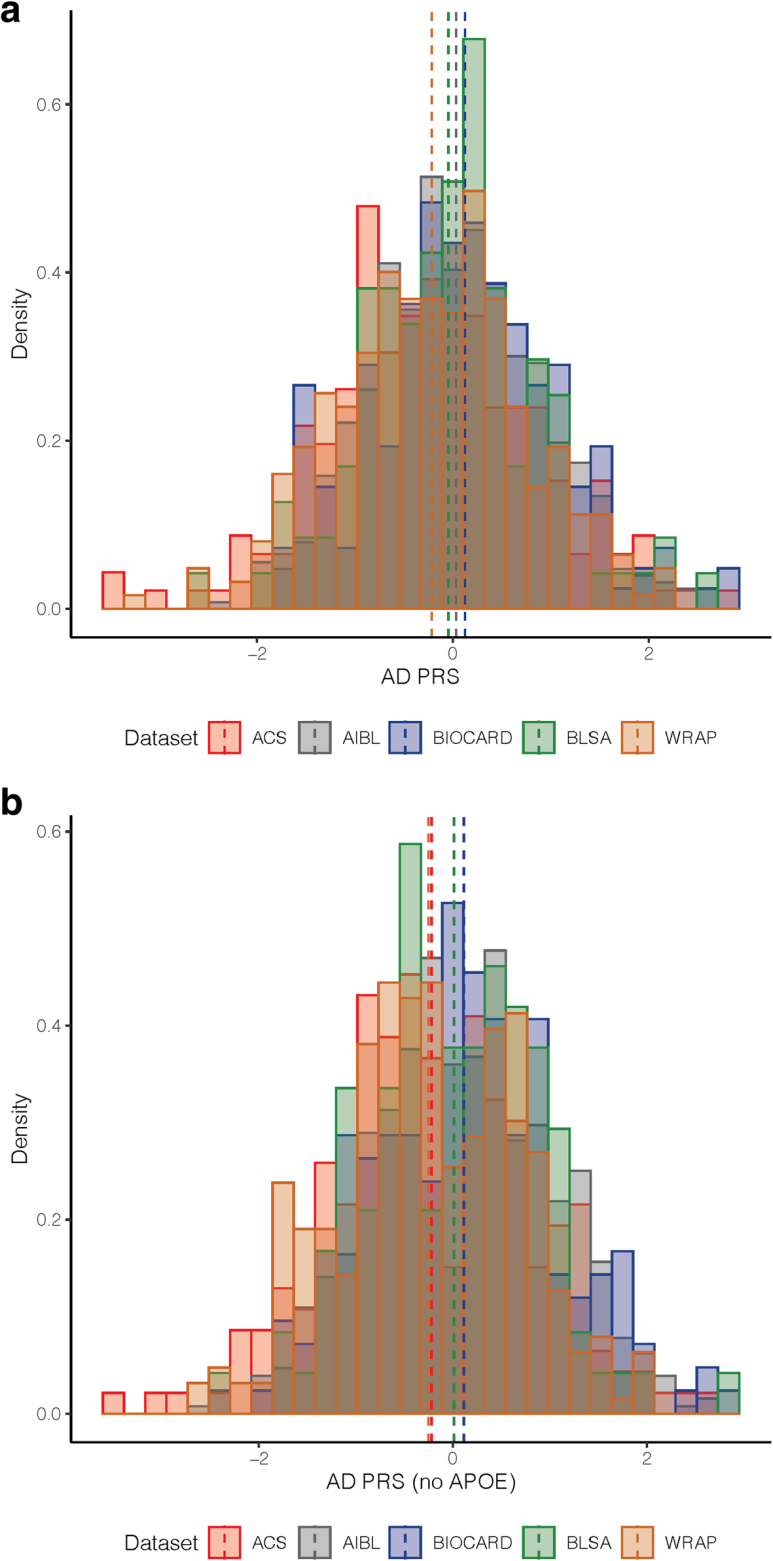
Distribution of harmonized AD-PRS in the PAC cohorts. Distributions shown for AD-PRS_APOE_ (top) and AD-PRS_w/oAPOE_ (bottom) in each of the PAC cohorts. The dashed vertical lines indicate the mean for each cohort. Note that the dashed lines for ACS and WRAP slightly overlap (top), as do the dashed lines for AIBL and BIOCARD (bottom)

Baseline characteristics of participants included in the analyses are shown in Table 1, for the *APOE* and AD-PRS analyses (for baseline characteristics by cohort, see Supplementary Tables 3 and 4, Additional File 1). On average, participants were late middle-aged at baseline, highly educated, approximately one-third *APOE-ε4* carriers, and have undergone approximately 10 years of follow-up (maximum = 28.8 years). In each cohort, years of education was moderately correlated with literacy test scores (all *r* > 0.26, all *p* < 0.001). As shown in Fig. 1, the distributions of the harmonized AD-PRS from the five cohorts were on the same scale and aligned across datasets. While the scores were harmonized with identical weights and variants used across cohorts, we did note a difference in the observed AD-PRS across datasets (*F*(4, 1379) = 5.88, *p* < 0.001) with ACS and WRAP presenting with a slightly lower AD-PRS than the other cohorts. This difference was observed for the score with and without *APOE* (*p* < 0.01). As expected, there were no direct associations between the AD genetic risk factors (i.e., *APOE-ε4, APOE-ε2*, AD-PRS_w/oAPOE_, or AD-PRS_APOE_) and the CR index score (all *p* > 0.34).

**Table 1:** Participant characteristics at baseline. Values reflect mean (SD) unless otherwise indicated

	Participants in *APOE* analyses	Participants in AD-PRS analyses
*N*	1819	1175
Age at baseline cognitive assessment	63.90 (10.13)	62.54 (10.08)
Female sex, *N* (%)	1069 (59%)	711 (61%)
Years of education	14.99 (3.20)	15.89 (3.25)
CR index score	0.03 (0.83)	0.00 (0.84)
*APOE-ε3* carriers, *N* (%)	1011 (56%)	668 (57%)
*APOE-ε2* carriers, *N* (%)	226 (12%)	133 (11%)
*APOE-ε4* carriers, *N* (%)	582 (32%)	374 (32%)
Global factor score	0.07 (0.95)	0.20 (0.92)
Episodic memory factor score ^a^	0.02 (0.97)	0.09 (0.98)
Executive function factor score ^b^	0.09 (0.91)	0.14 (0.88)
Number of cognitive scores over time	6.55 (4.13)	7.63 (3.84)
Years between baseline and last cognitive score [range]	9.85 (6.24) [0–28.8]	11.78 (5.17) [0–28.8]

^a^ *N APOE* analyses of memory factor score = 1813

^b^ *N APOE* analyses of executive function factor score = 1816

### CR index score, *APOE* genotypes, and cognitive change

Results for the *APOE* analyses are shown in Table 2. Higher CR index scores were associated with better baseline cognitive performance for all cognitive factor scores (all *p* < 0.001), but not with rate of change in cognition over time. *APOE-ε4* genotype was associated with greater rates of cognitive decline in all cognitive factor scores over time (all *p* < 0.001), whereas *APOE-ε2* genotype was not associated with cognitive performance (see Supplementary Figure 1, Additional File 1 for spaghetti plots of participant trajectories by AD genetic risk profiles).

**Table 2:** Mixed-effects model results for *APOE* genetic status and CR in relationship to cognitive trajectories (*N*=1819)

	Global factor score			Memory factor score			Executive function factor score		
	Estimate (95% CI)	*p*-value	Effect size	Estimate (95% CI)	*p*-value	Effect size	Estimate (95% CI)	*p*-value	Effect size
Time	− 0.002 (− 0.017, 0.013)	0.76	-	0.028 (0.009, 0.047)	0.004 **	-	− 0.023 (− 0.039, − 0.008)	0.003 **	-
Time^2^	− 0.004 (− 0.004, − 0.004)	< 0.001 ***	-	− 0.003 (− 0.003, − 0.002)	< 0.001 ***	-	− 0.004 (− 0.005, − 0.004)	< 0.001 ***	-
CR index	0.381 (0.330, 0.432)	< 0.001 ***	0.43	0.327 (0.268, 0.386)	< 0.001 ***	0.38	0.356 (0.300, 0.411)	< 0.001 ***	0.42
*APOE-ε2*	− 0.015 (− 0.117, 0.087)	0.77	0.02	− 0.029 (− 0.146, 0.088)	0.63	0.03	− 0.039 (− 0.150, 0.072)	0.49	0.05
*APOE-ε4*	− 0.012 (− 0.085, 0.061)	0.74	0.01	− 0.002 (− 0.085, 0.081)	0.96	0.00	− 0.028 (− 0.107, 0.050)	0.48	0.03
CR index × time	0.002 (− 0.005, 0.009)	0.53	0.03	0.003 (− 0.004, 0.011)	0.39	0.05	− 0.001 (− 0.007, 0.005)	0.83	0.01
*APOE-ε2* × time	0.005 (− 0.008, 0.018)	0.46	0.07	0.003 (− 0.012, 0.017)	0.71	0.04	0.004 (− 0.007, 0.016)	0.47	0.07
*APOE-ε4* × time	− 0.022 (− 0.031, − 0.012)	< 0.001 ***	0.30	− 0.024 (− 0.035, − 0.014)	< 0.001 ***	0.33	− 0.014 (− 0.023, − 0.006)	< 0.001 ***	0.24
CR index × *APOE-ε2*	− 0.007 (− 0.136, 0.123)	0.92	0.01	0.012 (− 0.136, 0.159)	0.88	0.01	− 0.020 (− 0.160, 0.120)	0.78	0.02
CR index × *APOE-ε4*	− 0.096 (− 0.185, − 0.008)	0.03 *	0.11	− 0.052 (− 0.153, 0.049)	0.31	0.06	− 0.086 (− 0.182, 0.009)	0.08	0.10
CR index × *APOE-ε2* × time	0.008 (− 0.008, 0.024)	0.33	0.11	0.011 (− 0.007, 0.030)	0.22	0.16	0.008 (− 0.007, 0.022)	0.30	0.12
CR index × *APOE-ε4* × time	0.012 (0.000, 0.023)	0.04 *	0.16	0.016 (0.003, 0.029)	0.01 *	0.22	0.007 (− 0.003, 0.017)	0.16	0.12

Separate models were estimated for the global, episodic memory, and executive function factor scores. Results of the full models including the 3-way interaction terms are shown; the patterns of results shown were the same when non-significant 3-way interaction terms were excluded. Models were additionally adjusted for baseline age, sex and cohort (ACS, AIBL, BIOCARD, BLSA, WRAP), and their interactions with time. ***p < 0.001; **p < 0.01; *p < 0.05. In all models, the coefficients for time reflect the starting trend of the trajectories (time = 0) whereas the coefficients for time^2^ reflect the change in trend over time

**Fig. 2:**
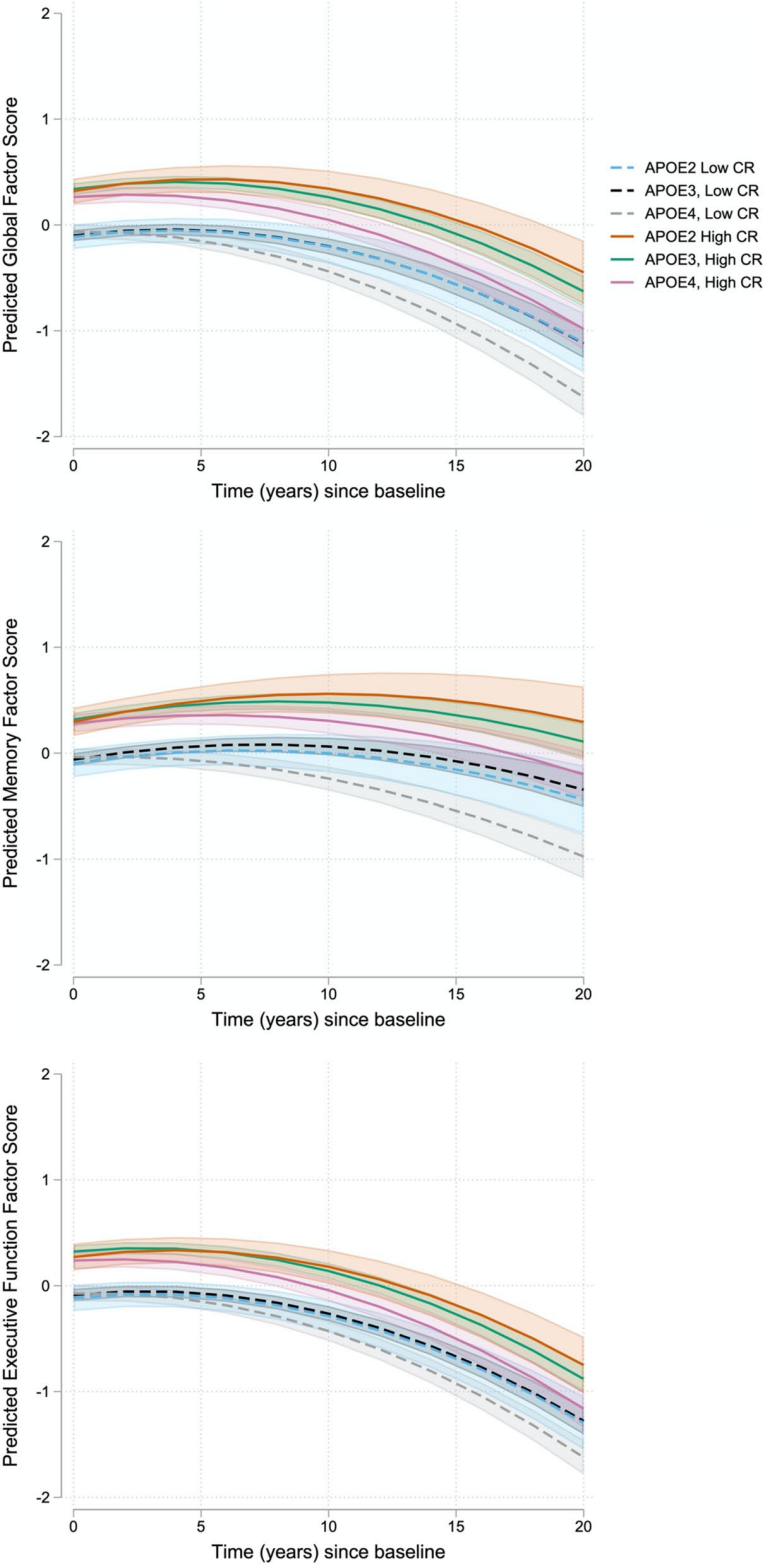
Estimates of longitudinal cognitive change based on *APOE* genetic status and level of cognitive reserve. Estimated cognitive change (95% CI) shown for *APOE* genetic status (*APOE-ε2*, *APOE-ε3*, *APOE-ε4*) and high vs. low CR index scores, represented by the 25th and 75th percentiles for illustration purposes. Sample means were used in the estimation of all other covariates. Cognitive change is shown separately for global (top), memory (middle), and executive function (bottom) factor scores based on the full models, as shown in Table 2. The CR index × *APOE-ε4* × time interactions were significant for the global and memory factor scores, but not for the executive function factor score

There were significant interactions between the CR index score and *APOE-ε4* genetic status. For the global factor score, there was a significant CR index × *APOE-ε4* interaction (*p* = 0.03), indicating that *APOE-ε4* carriers with higher CR index scores had lower baseline cognitive performance, relative to *APOE-ε3*. Of primary interest, there were significant 3-way CR index score × *APOE-ε4* × time interactions (both *p* ≤ 0.04) for the global and memory scores: model coefficients indicate that the negative effect of *APOE-ε4* genotype on global and memory score decline was attenuated among individuals with higher CR index scores (Fig. 2).

Across all models, there were significant main effects of age and sex, and age × time interactions (all *p* < 0.001) for all cognitive factor scores: baseline cognitive performance was lower among older participants and men, and older participants had greater rates of cognitive decline. There were also significant sex × time interactions (all *p* ≤ 0.04) for the executive function factor score only, indicating greater rates of decline among men compared to women.

### CR index score, AD-PRS and cognitive change

**Fig. 3:**
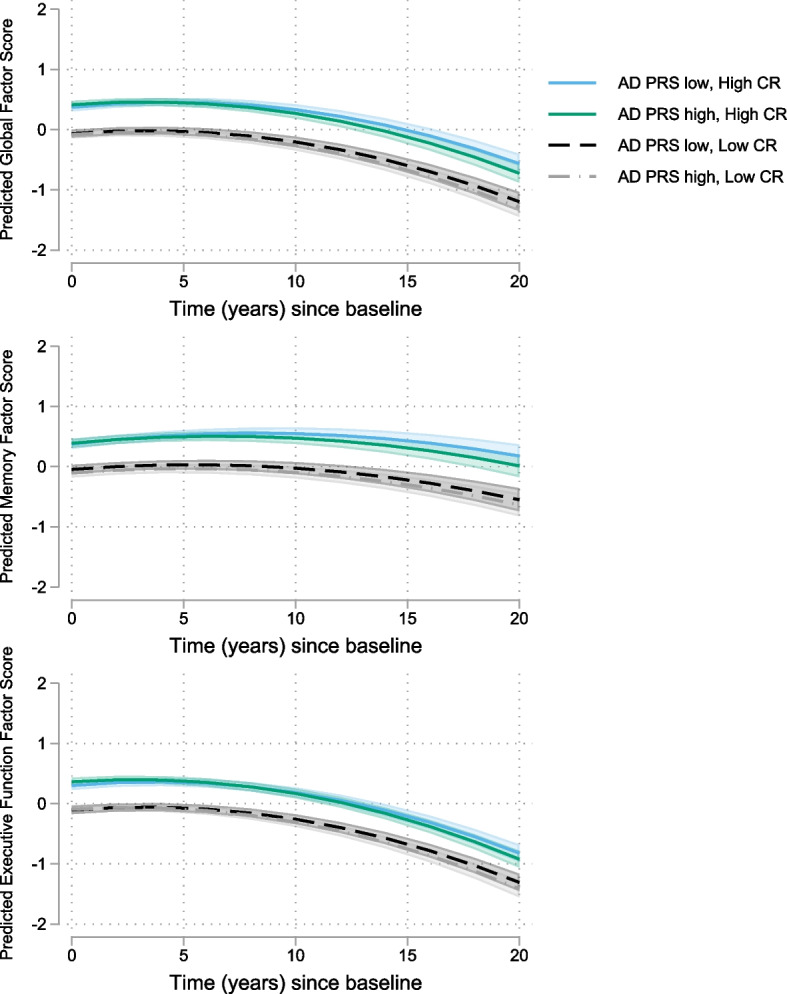
Estimates of longitudinal cognitive change based on AD-PRS_w/oAPOE_ and level of cognitive reserve. Estimated cognitive change (95% CI) shown for high vs. low AD-PRS_w/oAPOE_ and high vs. low CR index scores, represented by the 25th and 75th percentiles for illustration purposes. Sample means were used in the estimation of all other covariates. Cognitive change is shown separately for global (top), memory (middle), and executive function (bottom) factor scores based on the full models, as shown in Table 3. The AD-PRS_w/oAPOE_ × time interactions were significant for the global and executive function factor scores, but not for the memory factor score

Results for the AD-PRS_w/oAPOE_ analyses are shown in Table 3. As above, higher CR index scores were associated with better baseline cognitive performance for all cognitive factor scores (all *p* < 0.001). Higher CR index scores were also associated with more positive rates of change in memory factor scores (*p* = 0.02). Higher AD-PRS_w/oAPOE_ were associated with greater rates of decline in the global and executive function factor scores (all *p* ≤ 0.03), but not with memory decline (*p* = 0.21). However, there were no significant 3-way CR index × AD-PRS_w/oAPOE_ × time interactions (all *p* > 0.23), indicating that level of CR did not attenuate the effect of the AD-PRS_w/oAPOE_ on cognitive decline (Fig. 3). The results for AD-PRS_APOE_ were similar, except that the AD-PRS_APOE_ was associated with declines in all three cognitive factor scores (all *p* ≤ 0.02; Supplementary Table 5, Additional File 1). These results were essentially unchanged when cluster bootstrapping was applied (data not shown).

**Table 3:** Mixed-effects model results for AD-PRS_w/oAPOE_ and CR in relationship to cognitive trajectories (*N* = 1175)

	Global factor score			Memory factor score			Executive function factor score		
	Estimate (95% CI)	*p*-value	Effect size	Estimate (95% CI)	*p*-value	Effect size	Estimate (95% CI)	*p*-value	Effect size
Time	− 0.006 (− 0.023, 0.012)	0.52	-	0.014 (− 0.008, 0.037)	0.20	-	− 0.019 (− 0.037, − 0.001)	0.04 *	-
Time^2^	− 0.004 (− 0.005, − 0.004)	< 0.001 ***	-	− 0.003 (− 0.003, − 0.002)	< 0.001 ***	-	− 0.004 (− 0.005, − 0.004)	< 0.001 ***	-
CR index	0.331 (0.283, 0.379)	< 0.001 ***	0.38	0.326 (0.270, 0.382)	< 0.001 ***	0.37	0.307 (0.256, 0.357)	< 0.001 ***	0.37
AD-PRS_w/oAPOE_	0.009 (− 0.033, 0.051)	0.68	0.01	− 0.019 (− 0.068, 0.031)	0.46	0.02	0.027 (− 0.017, 0.072)	0.23	0.03
CR index × time	0.005 (− 0.001, 0.010)	0.10	0.07	0.008 (0.001, 0.015)	0.02 *	0.11	0.002 (− 0.003, 0.007)	0.43	0.03
AD-PRS_w/oAPOE_ × time	− 0.005 (− 0.010, 0.000)	0.03 *	0.08	− 0.004 (− 0.010, 0.002)	0.21	0.05	− 0.005 (− 0.010, − 0.001)	0.02 *	0.09
CR index × AD-PRS_w/oAPOE_	0.031 (− 0.020, 0.081)	0.23	0.04	0.040 (− 0.019, 0.099)	0.18	0.05	0.029 (− 0.024, 0.082)	0.28	0.04
CR index × AD-PRS_w/oAPOE_ × time	− 0.003 (− 0.009, 0.002)	0.25	0.05	− 0.004 (− 0.011, 0.003)	0.24	0.06	− 0.001 (− 0.007, 0.004)	0.61	0.02

Separate models were estimated for the global, episodic memory, and executive function factor scores. Results of the full models including the 3-way interaction terms are shown; the patterns of results shown were the same when non-significant 3-way interaction terms were excluded. Models were additionally adjusted for baseline age, sex and cohort (ACS, AIBL, BIOCARD, BLSA, WRAP), and their interactions with time. ***p < 0.001; **p < 0.01; *p < 0.05

### Sensitivity analyses

When participants who progressed from normal cognition to MCI or dementia were excluded from the models (see Supplementary Table 6, Additional File 1), the patterns of results were similar to those described above, with a few exceptions. In the *APOE* models (excluding *n* = 230 progressors), *APOE-ε4* genotype was associated with greater rates decline in the memory factor score (*p* = 0.02), but only marginally with declines in the global and executive function factor scores (both *p* < 0.08). There were no interactions between the CR index score and *APOE-ε4* genetic status. However, the 3-way CR index score × *APOE-ε4* × time interactions remained significant for the global and memory factor scores (both *p* ≤ 0.04), again indicating attenuated *APOE-ε4*-related global and memory score declines among individuals with higher CR index scores. In the AD-PRS_w/oAPOE_ models (excluding *n* = 178 progressors), there was no CR index score × time interaction for the memory factor score. Higher AD-PRS_w/oAPOE_ were associated with greater rates of decline only in the executive function factor score (*p* = 0.04) and not with declines in the global or memory factor scores (both *p* > 0.21). As above, there were no significant 3-way CR index × AD-PRS_w/oAPOE_ × time interactions (all *p* > 0.76). In the second set of sensitivity analyses, *z*-scored years of education and *z*-scored literacy scores were each significantly associated with better cognitive performance on all three factor scores, but only the years of education variable (not literacy test scores) attenuated *APOE-ε4*-related declines in the global and memory factor scores (Supplementary Table 7, Additional File 1).

All patterns of AD-PRS results were unchanged when *n* = 64 related individuals were excluded from the analyses and when covarying for population PCs (Supplementary Tables 8 and 9, Additional File 1). Additionally, the AD-PRS_w/oAPOE_ remained significantly associated with greater rates of decline in the global and executive function factor scores when terms for *APOE-ε4* genetic status were included as additional predictors (both *p* ≤ 0.03), whereas *APOE-ε4* genetic status was independently associated with greater rates of cognitive decline in all cognitive factor scores (all *p* ≤ 0.005; see Table 4).

**Table 4:** Mixed-effects model results for AD-PRS_w/oAPOE_, *APOE-ε4*, and CR in relationship to cognitive trajectories (*N* = 1175)

	Global factor score			Memory factor score			Executive function factor score		
	Estimate (95% CI)	*p*-value	Effect size	Estimate (95% CI)	*p*-value	Effect size	Estimate (95% CI)	*p*-value	Effect size
Time	0.003 (− 0.015, 0.020)	0.77	-	0.025 (0.003, 0.048)	0.03 *	-	− 0.014 (− 0.033, 0.004)	0.13	-
Time^2^	− 0.004 (− 0.005, − 0.004)	< 0.001 ***	-	− 0.003 (− 0.003, − 0.002)	< 0.001 ***	-	− 0.004 (− 0.005, − 0.004)	< 0.001 ***	-
CR index	0.330 (0.282, 0.378)	< 0.001 ***	0.38	0.325 (0.269, 0.381)	< 0.001 ***	0.37	0.307 (0.256, 0.357)	< 0.001 ***	0.37
AD-PRS_w/oAPOE_	0.009 (− 0.034, 0.051)	0.69	0.01	− 0.018 (− 0.068, 0.031)	0.46	0.02	0.028 (− 0.017, 0.072)	0.23	0.03
*APOE-ε4*	0.022 (− 0.065, 0.109)	0.62	0.03	0.040 (− 0.061, 0.141)	0.44	0.05	− 0.047 (− 0.138, 0.045)	0.32	0.06
CR index × time	0.005 (− 0.001, 0.010)	0.09	0.07	0.008 (0.002, 0.015)	0.01 *	0.11	0.002 (− 0.003, 0.007)	0.42	0.03
AD-PRS_w/oAPOE_ × time	− 0.005 (− 0.010, 0.000)	0.04 *	0.07	− 0.004 (− 0.009, 0.002)	0.21	0.05	− 0.005 (− 0.010, − 0.001)	0.02 *	0.09
*APOE-ε4* × time	− 0.025 (− 0.034, − 0.015)	< 0.001 ***	0.35	− 0.029 (− 0.040, − 0.017)	< 0.001 ***	0.38	− 0.012 (− 0.021, − 0.004)	0.005 **	0.21
CR index × AD-PRS_w/oAPOE_	0.031 (− 0.019, 0.082)	0.23	0.04	0.040 (− 0.018, 0.099)	0.18	0.05	0.029 (− 0.024, 0.082)	0.28	0.04
CR index × AD-PRS_w/oAPOE_ × time	− 0.004 (− 0.009, 0.002)	0.21	0.05	− 0.005 (− 0.011, 0.002)	0.20	0.06	− 0.002 (− 0.007, 0.004)	0.56	0.03

AD-PRS_w/oAPOE_ sensitivity analyses evaluating whether the relationship between the AD-PRS_w/oAPOE_ and cognitive trajectories remained when terms for *APOE-ε4* genetic status (i.e., *APOE-ε4* and *APOE-ε4* × time) were included as additional model predictors. Separate models were estimated for the global, episodic memory, and executive function factor scores. Results of the full models including the 3-way interaction terms are shown; the patterns of results shown were the same when non-significant 3-way interaction terms were excluded. Models were additionally adjusted for baseline age, sex and cohort (ACS, AIBL, BIOCARD, BLSA, WRAP), and their interactions with time. ***p < 0.001; **p < 0.01; *p < 0.05

## Discussion

This study examined the association between a CR index score and AD genetic risk factors in relationship to cognitive change among participants with normal cognition at baseline. There are several notable findings. First, higher CR index scores were consistently associated with better cognitive performance, whereas those at greater genetic risk for AD (based on either *APOE* genetic status or AD-PRS) demonstrated greater rates of cognitive decline, including among individuals who have remained cognitively normal over the course of follow-up. Second, higher levels of CR attenuated *APOE-ε4*-related declines in global cognition and memory. However, levels of CR did not attenuate *APOE-ε4*-related declines in executive function or AD-PRS-related cognitive decline. Of note, while *APOE-ε4* genetic status and AD-PRS_APOE_ were associated with declines in all cognitive domains examined here, AD-PRS_w/oAPOE_ was significantly associated with declines in executive function and global cognition but not episodic memory. Finally, *APOE-ε2* genetic status was unrelated to cognitive performance. These results suggest that AD genetic risk factors differentially impact cognitive trajectories among individuals with normal cognition at baseline. However, the impact of *APOE-ε4*, in particular, may be mitigated by lifestyle factors (such as CR) that are potentially modifiable.

### Interactions between level of CR and AD genetic risk

To our knowledge, no prior studies have evaluated interactions between CR proxy measures and both *APOE-ε4* and AD-PRS in the same group of participants. Our results suggest that higher levels of CR differentially mitigate *APOE-ε4*-related vs. AD-PRS-related cognitive decline, with the protective effect of CR being specific to *APOE-ε4*-related declines in episodic memory (but not executive function). This may suggest that higher levels of CR (e.g., education, literacy) reduce the impact of *APOE-ε4* on rates of change in episodic memory among older individuals. Given that middle-aged and older *APOE-ε4* carriers, on average, have more AD-related pathology than non-carriers due to an earlier onset of amyloid accumulation [55, 56], these results suggest that individuals with higher levels of CR may be better able to tolerate early AD-related brain changes. For example, individuals with higher levels of CR may have greater brain reserve (such as greater volume, cortical thickness, or microstructural integrity [57]) or more effectively utilize alternative behavioral strategies to compensate for declining memory processes [58, 59]. The mechanisms by which this occurs, however, remain unclear and future studies are needed to determine whether CR is protective against early AD vs. non-AD processes [60–62], especially given that level of CR also attenuated *APOE-ε4*-related memory declines in the subset of individuals who have remained cognitively normal over time. Of note, the effect sizes for these interactions were small (*d* = 0.16 and *d* = 0.22 for global cognition and memory, respectively), suggesting that the degree to which CR modifies *APOE-ε4*-associated declines is modest.

Only three prior studies have examined interactions between CR proxy measures and *APOE-ε4* genotype in relationship to longitudinal cognitive decline among individuals with normal cognition at baseline. Our results are consistent with those of Mayeux et al. [26], which reported greater *APOE-ε4*-related decline in an episodic memory factor score among individuals with lower levels of education, with similar effects not found for language and visuospatial/reasoning factor scores (total sample size = 563). However, other studies have reported different outcomes [27, 28]. For example, using data from WRAP, Koscik et al. [27] found only limited evidence of a relationship between *APOE-ε4* and decline on individual cognitive tests among cognitively normal middle-aged participants, and this relationship was not modified by literacy scores (total sample size = 1256). Discrepancies between studies may reflect differences in cohort age and the sensitivity of the cognitive tests used (e.g., individual tests vs. cognitive composite scores), or the fact that these interactions have small effect sizes that are difficult to detect in smaller samples.

In contrast to the *APOE-ε4* results, AD-PRS-related cognitive decline was not attenuated by level of CR. The one prior longitudinal study that has examined interactions between CR proxy measures and an AD-PRS on cognitive decline found only limited evidence for an interaction, as only one of eight CR proxy measures examined (reading books) was associated with reduced AD-PRS_APOE_-related cognitive decline [32]. The results of that study, however, are difficult to compare to our own because participants were of mixed clinical diagnoses at baseline (i.e., not restricted to individuals with normal cognition). Because AD-PRS reflect many genetic loci with heterogenous impacts on multiple molecular and neuropathological pathways [6, 63], it may be that levels of CR have effects on a subset of AD-PRS mechanistic pathways, but not others. Additional studies are needed to further examine these questions.

### AD genetic risk and cognitive decline

The AD-PRS_w/oAPOE_ was associated with global and executive function decline, but not episodic memory decline. This association was statistically significant when *APOE-ε4* genetic status was included as an additional model covariate. Additionally, in this latter set of models, *APOE-ε4* was independently associated with cognitive decline in all cognitive domains examined, and with larger effect sizes than the AD-PRS_w/oAPOE_ (see Table 4). This suggests that there may be different biological pathways that mediate AD-related cognitive decline across different cognitive domains, with some pathways having a greater impact on decline than others during the preclinical phase of AD. This likely reflects the fact that individuals at greater genetic risk (i.e., *APOE-ε4* carriers; higher AD-PRS scores) have more AD pathology (e.g., [11, 64–66]) which at least partially underlies the relationship between these genetic risk factors and cognitive decline [63, 67, 68].

While a number of prior studies have reported greater rates of cognitive decline among cognitively normal *APOE-ε4* carriers (for a review, see [60]), studies examining the relationship of AD-PRS with cognitive decline have been more limited. Analyses among non-demented and mixed diagnosis samples have reported relationships between AD-PRS_w/oAPOE_ and both executive function and episodic memory declines [12, 67, 69], whereas studies among individuals with normal cognition have been more mixed. Consistent with our results, Tan et al. [14] found significant associations between an AD-PRS_w/oAPOE_ and scores on several individual tests, including two attention/executive function tasks among cognitively normal individuals with AD pathology at autopsy. Additionally, a study among cognitively normal participants from AIBL found no association between an AD-PRS_w/oAPOE_ and decline on composite scores composed largely of episodic memory tasks, although an executive function composite was not examined (Porter et al., [66]). However, Gustavson et al. [70] found only limited evidence of an association between AD-PRS_w/oAPOE_ and cognitive decline among middle-aged cognitively normal individuals, as only one AD-PRS_w/oAPOE_ of several examined was associated with declines in episodic memory, and none of the PRS were associated with declines in an executive function factor score. While reasons for this mixed literature are not yet clear, these findings suggest that non-*APOE* AD risk genes have very subtle effects on cognitive change among individuals with normal cognition. This mixed literature may also be influenced by differences in methods used for PRS calculation, including the specific loci included. Additional studies are needed to further examine this question, and the possibility of domain-specific cognitive effects.

As noted above, greater genetic risk for AD was also associated with greater rates of cognitive decline among individuals who have remained cognitively normal over the course of follow-up. Specifically, *APOE-ε4* genetic status was significantly associated with declines in the episodic memory factor score, whereas the AD-PRS_w/oAPOE_ was associated with declines in the executive function factor score. This may suggest that AD genetic risk makes notable contributions to age-related declines in these domains, though future studies are needed to evaluate how these results change when accounting for individual differences in biomarkers of AD pathology.

### Level of CR and cognitive decline

In line with prior work, higher CR index scores were consistently associated with better overall cognitive performance, with less evidence of an association with change in cognition over time. This suggests that cognitive reserve confers protective effects largely by impacting level of cognitive performance across all levels of AD genetic risk (which may delay the age at which individuals reach clinically significant cognitive impairment), rather than reducing rates of cognitive decline ([71]; see also [25]). Identifying the neurobiological mechanisms of CR, and the ways by which these mechanisms modify age- and disease-related brain changes, is an important research priority.

### *APOE-ε2* and cognitive decline

Lastly, although the *APOE-ε2* genotype is associated with reduced risk of AD dementia [8, 9], we did not find a relationship between *APOE-ε2* genotype and cognitive trajectories in this sample of middle-aged and older cognitively normal individuals, either alone or in interaction with level of CR. To our knowledge, no prior studies have examined the interactions between *APOE-ε2* genotype and level of CR in relationship to longitudinal cognitive trajectories. Our results differ from a prior report from the BIOCARD study, which found that higher levels of CR were more protective in *APOE-ε2* carriers vs. non-carriers with respect to time to progression from normal cognition to MCI clinical symptom onset [29]. Reasons for these differing results are unclear but may be related to the outcome used (i.e., rate of change in cognition vs. time to onset of clinical symptoms). Furthermore, although prior studies have reported reduced rates of cognitive decline among *APOE-ε2* carriers, findings have been mixed, particularly in younger samples (for reviews, see [10, 72]). This suggests that the direct effects of *APOE-ε2* genotype on cognitive trajectories are more evident in older populations, among whom cognitive decline is more prevalent.

## Limitations

This study has limitations. First, participants were primarily White, well-educated, and several cohorts were enriched for a family history of dementia due to AD. Although the family history might be beneficial for examining the relationship between AD genetic risk factors and cognitive decline, it limits the generalizability of the findings to broader populations. Additionally, there were differences in characteristics between cohorts, including baseline age and level of AD genetic risk. While this in part reflects differences in cohort design (e.g., target age range; enrichment for family history of AD dementia), we cannot rule out the possibility of survival effects in the oldest participants, given *APOE-ε4* and AD-PRS effects and frequencies may vary with age (e.g., [8, 73–75]). Future studies are needed to evaluate whether the reported results differ between middle-aged and older adults, as well as by sex. Second, the AD-PRS models included fewer participants, and only 226/1819 participants (12%) were *APOE-ε2* carriers. We therefore cannot rule out the possibility that we were underpowered to detect 3-way interactions or relationships between *APOE-ε2* and cognition, despite the fact that the frequency of *APOE-ε2* carriers may be slightly higher than worldwide frequency estimates [76, 77]. Similarly, we cannot rule out the possibility that some of the effects reported here are false positives. For example, we used a *p* < .05 significance level for all analyses to examine complex inter-relationships between these variables and because few prior studies have examined these research questions among individuals with normal cognition. However, some of the interactions had modest levels of statistical significance and small effect sizes. Third, there are limitations related to the CR index score used in this study. The CR index score was composed of variables that are closely related to general intelligence, which may at least partly underlie associations with cognitive performance. These variables are likely also closely related to other factors, such as occupational complexity, socioeconomic status, and lifetime opportunities. Additional longitudinal studies are needed to replicate these findings in more diverse cohorts with a broader range of educational attainment, using other measures of CR (such as occupational complexity and engagement in cognitively stimulating activities), and while accounting for biomarkers of age- and disease-related brain changes.

## Conclusions

These findings suggest that higher levels of cognitive reserve attenuate *APOE-ε4*-related declines in global cognition and memory, but not *APOE-ε4*-related declines in executive function, or AD PRS-related cognitive decline. This raises the possibility that interventions targeting intellectual activities may disproportionately benefit *APOE-ε4* carriers and supports recent recommendations on the importance of prioritizing early-life education and maintaining a cognitively active lifestyle in mid-life and later [2].

## Supplementary Information

**Additional file 1: Supplementary Table 1.** Cognitive tests included in the harmonized cognitive factor scores, by cohort. **Supplementary Table 2. ***APOE* genotyping approaches used by each PAC cohort. **Supplementary Table 3.** Baseline participant characteristics, by cohort, for *APOE* analyses. Values reflect mean (SD) unless otherwise indicated. **Supplementary Table 4.** Baseline participant characteristics, by cohort, for AD-PRS analyses. Values reflect mean (SD) unless otherwise indicated. **Supplementary Table 5.** Mixed-effects model results for AD-PRS_APOE_ and CR in relationship to cognitive trajectories. **Supplementary Table 6.** Descriptive statistics by follow-up diagnosis and mixed-effects model results for AD genetic risk and CR in relationship to cognitive trajectories, excluding individuals who progressed from normal cognition to MCI or dementia. **Supplementary Table 7.** Mixed-effects model results for AD genetic risk and the components of the CR index score in relationship to cognitive trajectories. **Supplementary Table 8.** Mixed-effects model results for AD-PRS_w/oAPOE_ and CR in relationship to cognitive trajectories, a) excluding related individuals and b) covarying for population PCs. **Supplementary Table 9.** Mixed-effects model results for AD-PRS_APOE_ and CR in relationship to cognitive trajectories, a) excluding related individuals and b) covarying for population PCs. **Supplementary Figure 1.** Spaghetti plots illustrating participant trajectories and estimated cognitive change by AD genetic risk profiles.

## Data Availability

The plan is to archive the PAC datafiles at the National Archive of Computerized Data on Aging (NACDA). Investigators interested in accessing the data should contact the PAC Coordinating Center at Johns Hopkins University for details.

## Abbreviations

ACS: Adult Children Study
AIBL: Australian Imaging, Biomarker, and Lifestyle
AD: Alzheimer’s disease
AD-PRS: Alzheimer’s disease polygenic risk scores
*APOE*: Apolipoprotein-E (gene)
BIOCARD: Biomarkers of Cognitive Decline Among Normal Individuals
BLSA: Baltimore Longitudinal Study of Aging
CFA: Confirmatory factor analysis
CI: Confidence interval
CR: Cognitive reserve
CSF: Cerebrospinal fluid
IRT: Item response theory
MCI: Mild cognitive impairment
MRI: Magnetic resonance imaging
NHW: Non-Hispanic white
PAC: Preclinical AD Consortium
PC: Principal component
PET: Positron emission tomography
SD: Standard deviation
WRAP: Wisconsin Registry for Alzheimer’s Prevention
