# Supplementary material for: Alzheimer’s disease genetic risk and cognitive reserve in relationship to long-term cognitive trajectories among cognitively normal individuals
Source: Alzheimers Res Ther. 2023 Mar 28;15:66. doi: 10.1186/s13195-023-01206-9 (PMC10045505; doi:10.1186/s13195-023-01206-9)

**Additional File 1: Supplemental Materials**

**Supplementary Tables**

Supplementary Table 1. Cognitive tests included in the harmonized cognitive factor scores, by cohort.

Supplementary Table 2. *APOE* genotyping approaches used by each PAC cohort.

Supplementary Table 3. Baseline participant characteristics, by cohort, for *APOE* analyses. Values reflect mean (SD) unless otherwise indicated.

Supplementary Table 4. Baseline participant characteristics, by cohort, for AD-PRS analyses. Values reflect mean (SD) unless otherwise indicated.

Supplementary Table 5. Mixed-effects model results for AD-PRS_APOE_ and CR in relationship to cognitive trajectories.

Supplementary Table 6. Descriptive statistics by follow-up diagnosis and mixed-effects model results for AD genetic risk and CR in relationship to cognitive trajectories, excluding individuals who progressed from normal cognition to MCI or dementia.

Supplementary Table 7. Mixed-effects model results for AD genetic risk and the components of the CR index score in relationship to cognitive trajectories.

Supplementary Table 8. Mixed-effects model results for AD-PRS_w/oAPOE_ and CR in relationship to cognitive trajectories, a) excluding related individuals and b) covarying for population PCs.

Supplementary Table 9. Mixed-effects model results for AD-PRS_APOE_ and CR in relationship to cognitive trajectories, a) excluding related individuals and b) covarying for population PCs.

**Supplementary Figure**

Supplementary Figure 1. Spaghetti plots illustrating participant trajectories and estimated cognitive change by AD genetic risk profiles.

Supplementary Table 1. Cognitive tests included in the harmonized cognitive factor scores, by cohort.

| Factor Score(s) | | Cognitive Test | ACS | AIBL | BIOCARD | BLSA | WRAP |
| --- | --- | --- | --- | --- | --- | --- | --- |
| Global | Episodic Memory | Logical Memory IA - Immediate | x | x | x |  | x |
|  |  | Logical Memory IIA - Delayed | x | x | x |  | x |
|  |  | Logical Memory IB - Immediate |  |  | x |  | x |
|  |  | Logical Memory IIB - Delayed |  |  | x |  | x |
|  |  | Paired Associates I - Immediate ^a^ | x |  | x |  |  |
|  |  | Paired Associates I - Delayed |  |  | x |  |  |
|  |  | California Verbal Learning Test - Immediate Recall |  | x | x | x |  |
|  |  | California Verbal Learning Test - Short Delay Recall |  | x | x | x |  |
|  |  | California Verbal Learning Test - Long Delay Recall (Number correct) |  | x | x | x |  |
|  |  | California Verbal Learning Test - Recognition Discriminability |  | x | x |  |  |
|  |  | Auditory Verbal Learning Test - Total Recall |  |  |  |  | x |
|  |  | Auditory Verbal Learning Test - Delayed Recall |  |  |  |  | x |
|  |  | Buschke Selective Reminding Test | x |  |  |  |  |
|  |  | Rey Complex Figure - Draw, 30 Minute Delay |  | x | x |  |  |
|  |  | Rey Complex Figure - Recognition |  | x |  |  |  |
|  | Executive Function | Digit Symbol Substitution | x |  | x | x | x |
|  |  | Digit Symbol Coding |  | x |  |  |  |
|  |  | Trail Making Test - Part B | x |  | x | x | x |
|  |  | Digit Span Backward (trails correct) | x | x | x | x | x |
|  |  | Digit Span Backward (length) | x |  | x | x |  |
|  |  | Category Fluency, Animals | x | x | x | x | x |
|  |  | Category Fluency, Vegetables | x |  | x | x |  |
|  |  | Category Fluency, Names |  | x |  |  |  |
|  |  | Category Fluency, Fruit |  | x |  |  |  |
|  |  | Category Fluency, Furniture |  | x |  |  |  |
|  |  | Stroop Test - C/D |  | x |  |  |  |
|  |  | Mini-Mental State Examination | x | x | x | x | x |
|  |  | Boston Naming Test | x | x | x | x | x |
|  |  | Trail Making Test - Part A | x |  | x | x | x |
|  |  | Digit Span Forward (trials correct) | x | x | x | x | x |
|  |  | Digit Span Forward (length) | x |  | x | x |  |
|  |  | Letter Fluency, F |  | x | x | x |  |
|  |  | Letter Fluency, A |  | x | x | x |  |
|  |  | Letter Fluency, S |  | x | x | x |  |
|  |  | Block Design | x |  | x |  | x |
|  |  | Rey Complex Figure - Copy |  | x | x |  |  |
|  |  | Rey Complex Figure - Draw, 3 Minute Delay |  | x |  |  |  |
|  |  | Clock Drawing |  | x |  |  |  |
|  |  | Stroop Test - Dots |  | x |  |  |  |
|  |  | Stroop Test - Words |  | x |  |  |  |
|  |  | Stroop Test - Colors |  | x |  |  |  |
|  |  | WAIS-R Similarities |  |  | x |  |  |

^a^ For Paired Associates I, ACS used Wechsler Memory Scale – 3rd edition (WMS-III) and BIOCARD used WMS-R; these were therefore treated as different variables when creating harmonized factor scores.

Supplementary Table 2. *APOE* genotyping approaches used by each PAC cohort.

*APOE*alleles were determined either by direct genotyping (rs7412 and rs429358) or restriction isotyping (codon 112 and 158).

| **Cohort** | ***APOE* Assay** | **Citation** |
| --- | --- | --- |
| ACS | Direct genotyping | Talbot C et al. Protection against Alzheimer's disease with apoE epsilon 2. *Lancet*. 1994;343(8910):1432-1433. doi: 10.1016/s0140-6736(94)92557-7 |
| AIBL | Direct genotyping (Taqman) | Fowler C et al. Fifteen Years of the Australian Imaging, Biomarkers and Lifestyle (AIBL) Study: Progress and Observations from 2,359 Older Adults Spanning the Spectrum from Cognitive Normality to Alzheimer's Disease. *J Alzheimers Dis Rep*. 2021;5(1):443-468. Published 2021 Jun 3. doi: 10.3233/ADR-210005 |
| BLSA | Restriction Isotyping | Resnick SM et al. Changes in Aβ biomarkers and associations with APOE genotype in 2 longitudinal cohorts. *Neurobiol Aging*. 2015;36(8):2333-2339. doi: 10.1016/j.neurobiolaging.2015.04.001 |
| BIOCARD | Restriction Isotyping | Albert M et al. Cognitive changes preceding clinical symptom onset of mild cognitive impairment and relationship to ApoE genotype. *Curr Alzheimer Res*. 2014;11(8):773-784. doi: 10.2174/156720501108140910121920 |
| WRAP | Direct genotyping | Johnson SC et al. The Wisconsin Registry for Alzheimer's Prevention: A review of findings and current directions. *Alzheimers Dement (Amst)*. 2017;10:130-142. Published 2017 Dec 8. doi: 10.1016/j.dadm.2017.11.007 |

Supplementary Table 3. Baseline participant characteristics, by cohort, for *APOE* analyses. Values reflect mean (SD) unless otherwise indicated.

|  |  | ACS | AIBL | BIOCARD | BLSA | WRAP | *p*-value |
| --- | --- | --- | --- | --- | --- | --- | --- |
| *N* | | 205 | 839 | 266 | 193 | 316 |  |
| Age at baseline cognitive assessment | | 62.02 (8.51) | 70.37 (6.00) | 57.05 (10.00) | 63.67 (11.24) | 54.11 (6.20) | < 0.001 |
| Female sex, N (%) | | 128 (62%) | 469 (56%) | 159 (60%) | 96 (50%) | 217 (69%) | < 0.001 |
| Years of education | | 16.27 (2.40) | 13.13 (3.09) | 17.04 (2.39) | 16.98 (2.13) | 16.14 (2.19) | < 0.001 |
| CR index score | | -0.08 (0.84) | 0.08 (0.83) | 0.09 (0.85) | 0.07 (0.77) | -0.11 (0.84) | 0.002 |
| *APOE-ε3* carriers, N (%) | | 100 (49%) | 492 (59%) | 145 (55%) | 111 (58%) | 163 (52%) | 0.05 |
| *APOE-ε2* carriers, N (%) | | 32 (16%) | 111 (13%) | 29 (11%) | 24 (12%) | 30 (9%) | 0.24 |
| *APOE-ε4* carriers, N (%) | | 73 (36%) | 236 (28%) | 92 (35%) | 58 (30%) | 123 (39%) | 0.004 |
| Global factor score | | 0.35 (0.78) | -0.28 (0.90) | 0.21 (0.84) | -0.20 (1.01) | 0.89 (0.55) | < 0.001 |
| Memory factor score | | -0.06 (1.35) | -0.14 (0.85) | 0.32 (0.89) | 0.14 (0.94) | 0.19 (0.99) | < 0.001 |
| Executive function factor score | | 0.43 (0.98) | -0.12 (0.90) | 0.20 (0.93) | -0.19 (0.91) | 0.49 (0.63) | < 0.001 |
| Number of cognitive scores over time | | 6.27 (2.92) | 4.87 (2.89) | 10.73 (4.03) | 10.69 (5.89) | 5.16 (1.02) | < 0.001 |
| Years between baseline and last cognitive score | | 8.32 (3.66) | 6.03 (4.40) | 15.27 (4.96) | 17.12 (7.65) | 11.96 (2.49) | < 0.001 |

Differences in baseline participant characteristics across cohorts assessed using a global *F* test for continuous variables or a global *χ^2^* test for categorical variables, as indicated by the *p*-value column in the table. Global tests were used to protect against false-positive results by examining the variability across all groups simultaneously.

Some of the differences in cohort characteristics, such as baseline age and *APOE-ε4* genetic status, reflect differences in study design. For example, the proportion of *APOE-ε4* carriers in AIBL is in line with the general population, whereas the three cohorts (ACS, BIOCARD, WRAP) with an overrepresentation of *APOE-ε4* carriers were enriched for a family history of Alzheimer’s disease dementia, by design. Additionally, these three cohorts have younger baseline ages because these studies enrolled individuals who were largely middle-aged at baseline, also by design. To help adjust for differences across cohorts, all mixed effect models included separate indicators for each cohort and cohort x time interaction terms.

Supplementary Table 4. Baseline participant characteristics, by cohort, for AD-PRS analyses. Values reflect mean (SD) unless otherwise indicated.

|  |  | ACS | AIBL | BIOCARD | BLSA | WRAP | *p*-value |
| --- | --- | --- | --- | --- | --- | --- | --- |
| *N* | | 135 | 453 | 190 | 109 | 288 |  |
| Age at baseline cognitive assessment | | 61.89 (8.88) | 70.27 (6.10) | 57.20 (9.48) | 62.25 (10.43) | 54.32 (6.24) | < 0.001 |
| Female sex, N (%) | | 86 (64%) | 248 (55%) | 117 (62%) | 60 (55%) | 200 (69%) | 0.001 |
| Years of education | | 16.26 (2.45) | 12.38 (2.92) | 16.99 (2.43) | 16.74 (2.10) | 16.11 (2.22) | < 0.001 |
| CR index score | | -0.07 (0.84) | 0.01 (0.85) | 0.14 (0.85) | 0.08 (0.77) | -0.11 (0.84) | 0.018 |
| *APOE-ε3* carriers, N (%) | | 67 (50%) | 276 (61%) | 109 (57%) | 71 (65%) | 145 (50%) | 0.007 |
| *APOE-ε2* carriers, N (%) | | 17 (13%) | 62 (14%) | 17 (9%) | 9 (8%) | 28 (10%) | 0.234 |
| *APOE-ε4* carriers, N (%) | | 51 (38%) | 115 (25%) | 64 (34%) | 29 (27%) | 115 (40%) | < 0.001 |
| Global factor score | | 0.37 (0.73) | -0.29 (0.91) | 0.26 (0.82) | 0.10 (0.82) | 0.90 (0.55) | < 0.001 |
| Memory factor score | | -0.04 (1.29) | -0.12 (0.87) | 0.36 (0.88) | 0.34 (0.91) | 0.22 (0.99) | < 0.001 |
| Executive cognitive factor score | | 0.51 (0.93) | -0.20 (0.88) | 0.25 (0.91) | 0.03 (0.75) | 0.48 (0.64) | < 0.001 |
| Number of cognitive scores over time | | 6.46 (2.86) | 6.72 (2.19) | 11.33 (3.84) | 12.63 (5.73) | 5.26 (0.88) | < 0.001 |
| Years between baseline and last cognitive score | | 8.54 (3.29) | 8.85 (3.26) | 15.99 (4.32) | 19.61 (6.74) | 12.12 (2.26) | < 0.001 |

Differences in baseline participant characteristics across cohorts assessed using a global *F* test for continuous variables or a global *χ^2^* test for categorical variables, as indicated by the *p*-value column in the table. Global tests were used to protect against false-positive results by examining the variability across all groups simultaneously.

Supplementary Table 5. Mixed-effects model results for AD-PRS_APOE_ and CR in relationship to cognitive trajectories.

|  | Global factor score | | Memory factor score | | Executive function factor score | |
| --- | --- | --- | --- | --- | --- | --- |
|  | Estimate (SE) | *p*-value | Estimate (SE) | *p*-value | Estimate (SE) | *p*-value |
| Time | -0.007 (0.009) | 0.41 | 0.013 (0.011) | 0.24 | -0.019 (0.009) | 0.04 * |
| Time^2^ | -0.004 (0.0002) | < 0.001 *** | -0.003 (0.0002) | < 0.001 *** | -0.004 (0.0002) | < 0.001 *** |
| CR index | 0.331 (0.025) | < 0.001 *** | 0.326 (0.029) | < 0.001 *** | 0.307 (0.026) | < 0.001 *** |
| AD-PRS_APOE_ | 0.007 (0.022) | 0.74 | -0.023 (0.025) | 0.36 | 0.019 (0.023) | 0.40 |
| CR index x time | 0.005 (0.003) | 0.10 | 0.008 (0.003) | 0.02 * | 0.002 (0.003) | 0.44 |
| AD-PRS_APOE_ x time | -0.009 (0.002) | < 0.001 *** | -0.007 (0.003) | 0.02 * | -0.007 (0.002) | 0.002 ** |
| CR index x AD-PRS_APOE_ | 0.004 (0.025) | 0.88 | 0.027 (0.030) | 0.35 | 0.009 (0.027) | 0.73 |
| CR index x AD-PRS_APOE_ x time | -0.0004 (0.003) | 0.87 | -0.0003 (0.004) | 0.93 | 0.0004 (0.003) | 0.87 |

Separate models were estimated for the global, episodic memory, and executive function factor scores. Results of the full models including the 3-way interaction terms are shown; the patterns of results shown were the same when non-significant 3-way interaction terms were excluded. Models were additionally adjusted for baseline age, sex and cohort (ACS, AIBL, BIOCARD, BLSA, WRAP), and their interactions with time. ****p* < 0.001; ***p* < 0.01; **p* < 0.05.

Supplementary Table 6. Descriptive statistics by follow-up diagnosis and mixed-effects model results for AD genetic risk and CR in relationship to cognitive trajectories, excluding individuals who progressed from normal cognition to MCI or dementia.

Supplementary Table 6a. Descriptive statistics by follow-up diagnosis (i.e., remain cognitively normal vs. progress to MCI or dementia), for participants included in the *APOE* analyses.

|  |  | Remain normal | Progress to MCI or dementia | *p*-value |
| --- | --- | --- | --- | --- |
| *N* | | 1589 | 230 |  |
| Age at baseline cognitive assessment | | 63.5 (10.14) | 67.1 (9.47) | < 0.001 |
| Female sex, N (%) | | 964 (60.7%) | 105 (45.7%) | < 0.001 |
| Years of education | | 14.98 (3.14) | 15.04 (3.57) | 0.79 |
| CR index score | | 0.05 (0.82) | -0.10 (0.94) | 0.01 |
| *APOE-ε3* carriers, N (%) | | 901 (56.7%) | 110 (47.8%) | 0.01 |
| *APOE-ε2* carriers, N (%) | | 196 (12.3%) | 30 (13.0%) | 0.76 |
| *APOE-ε4* carriers, N (%) | | 492 (31.0%) | 90 (39.1%) | 0.01 |
| Global factor score | | 0.16 (0.91) | -0.51 (0.96) | < 0.001 |
| Episodic memory factor score | | 0.05 (0.87) | -0.43 (0.92) | < 0.001 |
| Executive function factor score | | 0.16 (0.89) | -0.44 (0.88) | < 0.001 |
| Number of cognitive scores over time | | 6.29 (3.93) | 9.20 (4.54) | < 0.001 |
| Years between baseline and last cognitive score [range] | | 9.57 (6.11) [0-28.1] | 12.70 (6.12) [1.3-28.3] | < 0.001 |

Supplementary Table 6b. Mixed-effects model results for *APOE* and CR in relationship to cognitive trajectories, excluding *n* = 230 individuals who progressed from normal cognition to MCI or dementia.

|  | Global factor score | | Memory factor score | | Executive function factor score | |
| --- | --- | --- | --- | --- | --- | --- |
|  | Estimate (SE) | *p*-value | Estimate (SE) | *p*-value | Estimate (SE) | *p*-value |
| Time | 0.012 (0.006) | 0.03 * | 0.031 (0.008) | < 0.001 *** | -0.010 (0.007) | 0.17 |
| Time^2^ | -0.003 (0.0002) | < 0.001 *** | -0.002 (0.0002) | < 0.001 *** | -0.003 (0.0002) | < 0.001 *** |
| CR index | 0.287 (0.025) | < 0.001 *** | 0.240 (0.030) | < 0.001 *** | 0.293 (0.029) | < 0.001 *** |
| APOE2 | -0.001 (0.052) | 0.99 | -0.009 (0.061) | 0.89 | -0.038 (0.058) | 0.51 |
| CR index x APOE2 | 0.064 (0.067) | 0.34 | 0.074 (0.078) | 0.35 | 0.013 (0.074) | 0.86 |
| CR index x time | -0.001 (0.002) | 0.68 | 0.000 (0.003) | 0.89 | -0.003 (0.003) | 0.34 |
| APOE2 x time | 0.002 (0.005) | 0.62 | -0.002 (0.006) | 0.74 | 0.005 (0.005) | 0.35 |
| CR index x APOE2 x time | 0.006 (0.006) | 0.35 | 0.014 (0.008) | 0.08 | 0.005 (0.006) | 0.46 |
| APOE4 | -0.018 (0.037) | 0.63 | 0.006 (0.043) | 0.89 | 0.003 (0.041) | 0.93 |
| CR index x APOE4 | 0.023 (0.045) | 0.61 | 0.084 (0.053) | 0.11 | -0.025 (0.050) | 0.62 |
| APOE4 x time | -0.006 (0.003) | 0.07 | -0.011 (0.005) | 0.02 * | -0.007 (0.004) | 0.06 |
| CR index x APOE4 x time | 0.009 (0.004) | 0.04 * | 0.014 (0.006) | 0.02 * | 0.007 (0.004) | 0.11 |

Separate models were estimated for the global, episodic memory, and executive function factor scores. Models were additionally adjusted for baseline age, sex and cohort (ACS, AIBL, BIOCARD, BLSA, WRAP), and their interactions with time. ****p* < 0.001; ***p* < 0.01; **p* < 0.05.

Supplementary Table 6c. Descriptive statistics by follow-up diagnosis (i.e., remain cognitively normal vs. progress to MCI or dementia), for participants included in the AD-PRS analyses.

|  |  | Remain normal | Progress to MCI or dementia | *p*-value |
| --- | --- | --- | --- | --- |
| *N* | | 997 | 178 |  |
| Age at baseline cognitive assessment | | 61.7 (10.01) | 67.0 (9.28) | < 0.001 |
| Female sex, N (%) | | 630 (63.2%) | 81 (45.5%) | < 0.001 |
| Years of education | | 14.93 (3.20) | 14.71 (3.54) | 0.41 |
| CR index score | | 0.01 (0.83) | -0.09 (0.92) | 0.14 |
| *APOE-ε3* carriers, N (%) | | 576 (57.8%) | 92 (51.7%) | 0.13 |
| *APOE-ε2* carriers, N (%) | | 115 (11.5%) | 18 (10.1%) | 0.58 |
| *APOE-ε4* carriers, N (%) | | 306 (30.7%) | 68 (38.2%) | 0.05 |
| Global factor score | | 0.32 (0.87) | -0.42 (0.91) | < 0.001 |
| Episodic memory factor score | | 0.15 (0.88) | -0.40 (0.93) | < 0.001 |
| Executive function factor score | | 0.22 (0.86) | -0.37 (0.85) | < 0.001 |
| Number of cognitive scores over time | | 7.32 (3.57) | 9.65 (4.67) | < 0.001 |
| Years between baseline and last cognitive score [range] | | 11.54 (4.91) [0-28.8] | 13.30 (6.21) [1.4-28.3] | < 0.001 |

Supplementary Table 6d. Mixed-effects model results for AD-PRS_w/oAPOE_ and CR in relationship to cognitive trajectories, excluding *n* = 178 individuals who progressed from normal cognition to MCI or dementia.

|  | Global factor score | | Memory factor score | | Executive function factor score | |
| --- | --- | --- | --- | --- | --- | --- |
|  | Estimate (SE) | *p*-value | Estimate (SE) | *p*-value | Estimate (SE) | *p*-value |
| Time | 0.015 (0.007) | 0.03 * | 0.023 (0.010) | 0.02 * | -0.005 (0.008) | 0.53 |
| Time^2^ | -0.003 (0.0002) | < 0.001 *** | -0.001 (0.0003) | < 0.001 *** | -0.003 (0.0002) | < 0.001 *** |
| CR index | 0.275 (0.024) | < 0.001 *** | 0.283 (0.029) | < 0.001 *** | 0.277 (0.026) | < 0.001 *** |
| AD-PRS_w/oAPOE_ | 0.003 (0.022) | 0.87 | -0.038 (0.027) | 0.16 | 0.031 (0.024) | 0.20 |
| CR index x AD-PRS_w/oAPOE_ | 0.019 (0.027) | 0.48 | 0.023 (0.032) | 0.48 | 0.038 (0.029) | 0.19 |
| CR index x time | 0.002 (0.002) | 0.43 | 0.004 (0.003) | 0.17 | 0.0001 (0.002) | 0.96 |
| AD-PRS_w/oAPOE_ x time | -0.002 (0.002) | 0.22 | -0.002 (0.003) | 0.47 | -0.004 (0.002) | 0.04 * |
| CR index x AD-PRS_w/oAPOE_ x time | -0.001 (0.002) | 0.77 | -0.001 (0.003) | 0.84 | 0.0003 (0.002) | 0.91 |

Separate models were estimated for the global, episodic memory, and executive function factor scores. Models were additionally adjusted for baseline age, sex and cohort (ACS, AIBL, BIOCARD, BLSA, WRAP), and their interactions with time. ****p* < 0.001; ***p* < 0.01; **p* < 0.05.

Supplementary Table 7. Mixed-effects model results for AD genetic risk and the components of the CR index score in relationship to cognitive trajectories.

To evaluate whether years and education and literacy scores make unique contributions to cognitive performance, we ran a series of sensitivity analyses in which the primary models (i.e., those reported in Tables 2 and 3 of the manuscript) were re-run using terms for z-scored education and z-scored literacy scores as simultaneous predictors of cognitive trajectories (in place of the terms for the CR index score). The results of the *APOE* and AD-PRS_w/oAPOE_ models are shown in Supplementary Tables 7a and 7b below, respectively. In all models, there were significant main effects of both the years of education and literacy variables, indicating that greater years of education and higher literacy scores were each independently associated with better cognitive performance on all three cognitive factor scores. Furthermore, within each cohort, the years of education and literacy scores were only moderately correlated (all *r* > 0.26 and < 0.52, all *p* < 0.001; see below). Together, this suggests that the education and literacy variables provide unique information related to cognitive performance. However, the three-way interaction between level of CR, *APOE-ε4*, and time was significant for the years of education variable, and not the literacy variable.

Correlations between the years of education and literacy test score variables, by cohort:

- ACS: *r* = 0.36, *p* < 0.0001
- AIBL: *r* = 0.41, *p* < 0.0001
- BIOCARD: *r* = 0.40, *p* < 0.0001
- BLSA: *r* = 0.27, *p* = 0.0001
- WRAP: *r* = 0.51, *p* < 0.0001

Supplementary Table 7a. Mixed-effects model results for *APOE,* z-scored years of education, and z-scored vocabulary scores in relationship to cognitive trajectories.

|  | Global factor score | | Memory factor score | | Executive function factor score | |
| --- | --- | --- | --- | --- | --- | --- |
|  | Estimate (SE) | *p*-value | Estimate (SE) | *p*-value | Estimate (SE) | *p*-value |
| Time | -0.001 (0.008) | 0.86 | 0.028 (0.010) | 0.004 ** | -0.022 (0.008) | 0.005 ** |
| Time^2^ | -0.004 (0.0001) | < 0.001 *** | -0.003 (0.0002) | < 0.001 *** | -0.004 (0.0002) | < 0.001 *** |
| Education | 0.085 (0.023) | < 0.001 *** | 0.071 (0.026) | 0.007 ** | 0.107 (0.025) | < 0.001 *** |
| Literacy | 0.260 (0.023) | < 0.001 *** | 0.206 (0.027) | < 0.001 *** | 0.223 (0.026) | < 0.001 *** |
| *APOE-ε2* | 0.006 (0.051) | 0.90 | -0.017 (0.058) | 0.77 | -0.024 (0.056) | 0.67 |
| *APOE-ε4* | -0.022 (0.036) | 0.54 | -0.002 (0.041) | 0.95 | -0.039 (0.040) | 0.32 |
| Education x time | -0.005 (0.003) | 0.10 | -0.003 (0.004) | 0.39 | -0.002 (0.003) | 0.46 |
| Literacy x time | 0.005 (0.003) | 0.10 | 0.005 (0.004) | 0.13 | 0.001 (0.003) | 0.86 |
| *APOE-ε2* x time | 0.007 (0.006) | 0.31 | 0.003 (0.007) | 0.66 | 0.004 (0.006) | 0.45 |
| *APOE-ε4* x time | -0.022 (0.005) | < 0.001 *** | -0.025 (0.005) | < 0.001 *** | -0.015 (0.004) | < 0.001 *** |
| Education x *APOE-ε2* | 0.087 (0.056) | 0.12 | 0.111 (0.063) | 0.08 | 0.070 (0.060) | 0.25 |
| Literacy x *APOE-ε2* | -0.009 (0.061) | 0.88 | -0.016 (0.069) | 0.82 | -0.029 (0.066) | 0.66 |
| Education x *APOE-ε4* | -0.032 (0.039) | 0.41 | 0.003 (0.044) | 0.94 | 0.004 (0.042) | 0.93 |
| Literacy x *APOE-ε4* | -0.018 (0.040) | 0.65 | 0.006 (0.046) | 0.90 | -0.081 (0.044) | 0.06 |
| Education x *APOE-ε2* x time | 0.003 (0.007) | 0.71 | 0.003 (0.008) | 0.73 | -0.004 (0.007) | 0.57 |
| Literacy x *APOE-ε2* x time | 0.012 (0.007) | 0.12 | 0.012 (0.008) | 0.17 | 0.014 (0.007) | 0.03 * |
| Education x *APOE-ε4* x time | 0.012 (0.005) | 0.02 * | 0.013 (0.006) | 0.02 * | 0.003 (0.005) | 0.49 |
| Literacy x *APOE-ε4* x time | 0.003 (0.005) | 0.60 | 0.005 (0.006) | 0.42 | 0.005 (0.005) | 0.25 |

Separate models were estimated for the global, episodic memory, and executive function factor scores. Models were additionally adjusted for baseline age, sex and cohort (ACS, AIBL, BIOCARD, BLSA, WRAP), and their interactions with time. ****p* < 0.001; ***p* < 0.01; **p* < 0.05.

Supplementary Table 7b. Mixed-effects model results for AD-PRS_w/oAPOE_, z-scored years of education, and z-scored vocabulary scores in relationship to cognitive trajectories.

|  | Global factor score | | Memory factor score | | Executive function factor score | |
| --- | --- | --- | --- | --- | --- | --- |
|  | Estimate (SE) | *p*-value | Estimate (SE) | *p*-value | Estimate (SE) | *p*-value |
| Time | -0.005 (0.009) | 0.57 | 0.014 (0.011) | 0.23 | -0.018 (0.009) | 0.05 |
| Time^2^ | -0.004 (0.0002) | < 0.001 *** | -0.003 (0.0002) | < 0.001 *** | -0.004 (0.0002) | < 0.001 *** |
| Education | 0.060 (0.023) | 0.009 ** | 0.077 (0.027) | 0.004 ** | 0.103 (0.024) | < 0.001 *** |
| Literacy | 0.249 (0.023) | < 0.001 *** | 0.211 (0.027) | < 0.001 *** | 0.191 (0.024) | < 0.001 *** |
| AD-PRS_w/oAPOE_ | 0.008 (0.021) | 0.72 | -0.004 (0.025) | 0.86 | 0.020 (0.023) | 0.36 |
| Education x time | -0.003 (0.003) | 0.32 | -0.001 (0.003) | 0.65 | -0.002 (0.002) | 0.39 |
| Literacy x time | 0.007 (0.003) | 0.02 * | 0.009 (0.003) | < 0.001 *** | 0.004 (0.002) | 0.13 |
| AD-PRS_w/oAPOE_ x time | -0.006 (0.002) | 0.02 * | -0.004 (0.003) | 0.22 | -0.005 (0.002) | 0.01 * |
| Education x AD-PRS_w/oAPOE_ | -0.001 (0.024) | 0.98 | -0.013 (0.028) | 0.65 | 0.013 (0.025) | 0.60 |
| Literacy x AD-PRS_w/oAPOE_ | 0.029 (0.023) | 0.22 | 0.015 (0.027) | 0.58 | 0.025 (0.025) | 0.31 |
| Education x AD-PRS_w/oAPOE_ x time | -0.002 (0.003) | 0.39 | 0.000 (0.003) | 0.93 | -0.001 (0.003) | 0.80 |
| Literacy x AD-PRS_w/oAPOE_ x time | 0.000 (0.003) | 0.87 | -0.004 (0.003) | 0.22 | -0.0004 (0.003) | 0.86 |

Separate models were estimated for the global, episodic memory, and executive function factor scores. Models were additionally adjusted for baseline age, sex and cohort (ACS, AIBL, BIOCARD, BLSA, WRAP), and their interactions with time. ****p* < 0.001; ***p* < 0.01; **p* < 0.05.

Supplementary Table 8. Mixed-effects model results for AD-PRS_w/oAPOE_ and CR in relationship to cognitive trajectories, a) excluding related individuals and b) covarying for population PCs.

Supplementary Table 8a. Mixed-effects model results for AD-PRS_w/oAPOE_ and CR in relationship to cognitive trajectories, excluding related individuals.

|  | Global factor score | | Memory factor score | | Executive function factor score | |
| --- | --- | --- | --- | --- | --- | --- |
|  | Estimate (SE) | *p*-value | Estimate (SE) | *p*-value | Estimate (SE) | *p*-value |
| Time | -0.005 (0.009) | 0.59 | 0.015 (0.011) | 0.19 | -0.018 (0.009) | 0.05 * |
| Time^2^ | -0.004 (0.0002) | < 0.001 *** | -0.003 (0.0002) | < 0.001 *** | -0.004 (0.0002) | < 0.001 *** |
| CR index | 0.309 (0.024) | < 0.001 *** | 0.288 (0.028) | < 0.001 *** | 0.295 (0.025) | < 0.001 *** |
| AD-PRS_w/oAPOE_ | -0.001 (0.021) | 0.95 | -0.011 (0.025) | 0.65 | 0.016 (0.022) | 0.47 |
| CR index x time | 0.004 (0.003) | 0.16 | 0.008 (0.003) | 0.02 * | 0.002 (0.003) | 0.53 |
| AD-PRS_w/oAPOE_ x time | -0.006 (0.002) | 0.01 * | -0.004 (0.003) | 0.17 | -0.006 (0.002) | 0.01 * |
| CR index x AD-PRS_w/oAPOE_ | 0.037 (0.025) | 0.14 | 0.009 (0.029) | 0.76 | 0.042 (0.026) | 0.11 |
| CR index x AD-PRS_w/oAPOE_ x time | -0.003 (0.003) | 0.39 | -0.004 (0.004) | 0.24 | -0.001 (0.003) | 0.71 |

AD-PRS_w/oAPOE_ sensitivity analyses excluding *n* = 64 related participants. Separate models were estimated for the global, episodic memory, and executive function factor scores. Models were additionally adjusted for baseline age, sex and cohort (ACS, AIBL, BIOCARD, BLSA, WRAP), and their interactions with time. ****p* < 0.001; ***p* < 0.01; **p* < 0.05.

Supplementary Table 8b. Mixed-effects model results for AD-PRS_w/oAPOE_ and CR in relationship to cognitive trajectories, excluding related individuals and covarying for population PCs.

Although analyses were restricted to NHW individuals, we note that results remained unchanged when performing sensitivity analyses including the first five population PCs as covariates, for ensuring results were not driven by any unmeasured population stratification due to genetic ancestry.

|  | Global factor score | | Memory factor score | | Executive function factor score | |
| --- | --- | --- | --- | --- | --- | --- |
|  | Estimate (SE) | *p*-value | Estimate (SE) | *p*-value | Estimate (SE) | *p*-value |
| Time | -0.006 (0.009) | 0.51 | 0.015 (0.012) | 0.21 | -0.019 (0.009) | 0.04 * |
| Time^2^ | -0.004 (0.0002) | < 0.001 *** | -0.003 (0.0003) | < 0.001 *** | -0.004 (0.0002) | < 0.001 *** |
| CR index | 0.303 (0.026) | < 0.001 *** | 0.310 (0.030) | < 0.001 *** | 0.293 (0.027) | < 0.001 *** |
| AD-PRS_w/oAPOE_ | 0.008 (0.023) | 0.74 | 0.006 (0.026) | 0.83 | 0.016 (0.024) | 0.50 |
| CR index x time | 0.004 (0.003) | 0.22 | 0.008 (0.004) | 0.03 * | -0.001 (0.003) | 0.84 |
| AD-PRS_w/oAPOE_ x time | -0.006 (0.003) | 0.02 * | -0.003 (0.003) | 0.26 | -0.006 (0.002) | 0.01 * |
| CR index x AD-PRS_w/oAPOE_ | 0.038 (0.026) | 0.14 | 0.017 (0.030) | 0.57 | 0.037 (0.028) | 0.18 |
| CR index x AD-PRS_w/oAPOE_ x time | -0.002 (0.003) | 0.45 | -0.004 (0.004) | 0.28 | -0.001 (0.003) | 0.66 |

Separate models were estimated for the global, episodic memory, and executive function factor scores. Models were additionally adjusted for baseline age, sex, and cohort (ACS, AIBL, BIOCARD, BLSA, WRAP), and their interactions with time, as well as the first five population PCs. ****p* < 0.001; ***p* < 0.01; **p* < 0.05.

Supplementary Table 9. Mixed-effects model results for AD-PRS_APOE_ and CR in relationship to cognitive trajectories, a) excluding related individuals and b) covarying for population PCs.

Supplementary Table 9a. Mixed-effects model results for AD-PRS_APOE_ and CR in relationship to cognitive trajectories, excluding related individuals.

|  | Global factor score | | Memory factor score | | Executive function factor score | |
| --- | --- | --- | --- | --- | --- | --- |
|  | Estimate (SE) | *p*-value | Estimate (SE) | *p*-value | Estimate (SE) | *p*-value |
| Time | -0.006 (0.009) | 0.48 | 0.014 (0.011) | 0.23 | -0.019 (0.009) | 0.04 * |
| Time^2^ | -0.004 (0.0002) | < 0.001 *** | -0.003 (0.0002) | < 0.001 *** | -0.004 (0.0002) | < 0.001 *** |
| CR index | 0.257 (0.020) | < 0.001 *** | 0.288 (0.028) | < 0.001 *** | 0.296 (0.025) | < 0.001 *** |
| AD-PRS_APOE_ | -0.001 (0.021) | 0.95 | -0.021 (0.025) | 0.39 | 0.010 (0.023) | 0.66 |
| CR index x time | 0.003 (0.002) | 0.17 | 0.008 (0.003) | 0.02 * | 0.002 (0.002) | 0.55 |
| AD-PRS_APOE_ x time | -0.010 (0.002) | < 0.001 *** | -0.007 (0.003) | 0.01 * | -0.007 (0.002) | 0.001 ** |
| CR index x AD-PRS_APOE_ | 0.013 (0.021) | 0.52 | 0.016 (0.029) | 0.58 | 0.021 (0.026) | 0.43 |
| CR index x AD-PRS_APOE_ x time | 0.000 (0.002) | 0.86 | 0.000 (0.004) | 0.98 | 0.001 (0.003) | 0.75 |

AD-PRS_APOE_ sensitivity analyses excluding *n* = 64 related participants. Separate models were estimated for the global, episodic memory, and executive function factor scores. Models were additionally adjusted for baseline age, sex and cohort (ACS, AIBL, BIOCARD, BLSA, WRAP), and their interactions with time. ****p* < 0.001; ***p* < 0.01; **p* < 0.05.

Supplementary Table 9b. Mixed-effects model results for AD-PRS_APOE_ and CR in relationship to cognitive trajectories, excluding related individuals and covarying for population PCs.

Although analyses were restricted to NHW individuals, we note that results remained unchanged when performing sensitivity analyses including the first five population PCs as covariates, for ensuring results were not driven by any unmeasured population stratification due to genetic ancestry.

|  | Global factor score | | Memory factor score | | Executive function factor score | |
| --- | --- | --- | --- | --- | --- | --- |
|  | Estimate (SE) | *p*-value | Estimate (SE) | *p*-value | Estimate (SE) | *p*-value |
| Time | -0.008 (0.009) | 0.39 | 0.013 (0.012) | 0.26 | -0.020 (0.009) | 0.03 * |
| Time^2^ | -0.004 (0.0002) | < 0.001 *** | -0.003 (0.0003) | < 0.001 *** | -0.004 (0.0002) | < 0.001 *** |
| CR index | 0.302 (0.026) | < 0.001 *** | 0.310 (0.030) | < 0.001 *** | 0.293 (0.027) | < 0.001 *** |
| AD-PRS_APOE_ | 0.000 (0.023) | 0.99 | -0.008 (0.026) | 0.76 | 0.005 (0.024) | 0.85 |
| CR index x time | 0.004 (0.003) | 0.24 | 0.008 (0.004) | 0.03 * | -0.001 (0.003) | 0.78 |
| AD-PRS_APOE_ x time | -0.010 (0.003) | < 0.001 *** | -0.006 (0.003) | 0.04 * | -0.007 (0.002) | 0.002 ** |
| CR index x AD-PRS_APOE_ | 0.013 (0.026) | 0.61 | 0.014 (0.030) | 0.63 | 0.017 (0.027) | 0.54 |
| CR index x AD-PRS_APOE_ x time | 0.001 (0.003) | 0.86 | 0.000 (0.004) | 0.99 | 0.000 (0.003) | 0.95 |

Separate models were estimated for the global, episodic memory, and executive function factor scores. Models were additionally adjusted for baseline age, sex, and cohort (ACS, AIBL, BIOCARD, BLSA, WRAP), and their interactions with time, as well as the first five population PCs. ****p* < 0.001; ***p* < 0.01; **p* < 0.05.

Supplementary Figure 1. Spaghetti plots illustrating participant trajectories and estimated cognitive change by AD genetic risk profiles.

Supplementary Figure 1a. Spaghetti plots illustrating participant trajectories and estimated cognitive change (95% CI) by *APOE* genetic status, including *APOE-ε2* (left), *APOE-ε3* (middle), and *APOE-ε4* (right). Cognitive change is shown separately for the global (top row), episodic memory (middle row), and executive function (bottom row) factor scores based on the full models, as shown in Table 2. Sample means were used in the estimation of all other covariates. The *APOE-ε4* x time interactions were significant for all three factor scores.


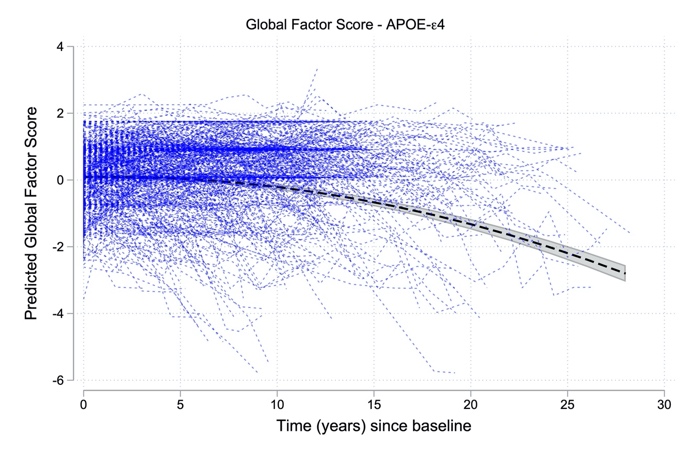

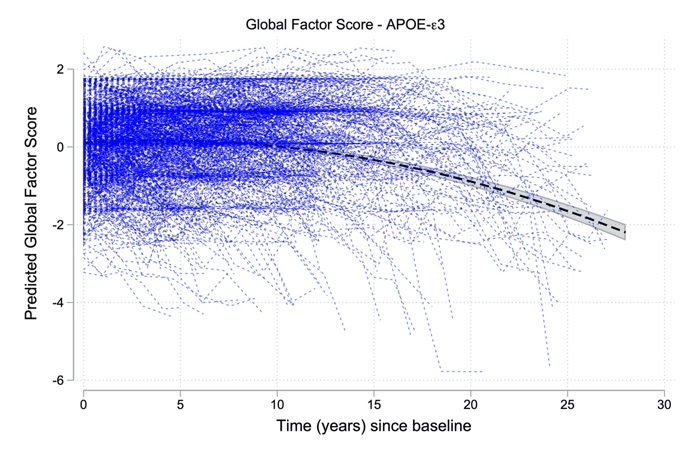


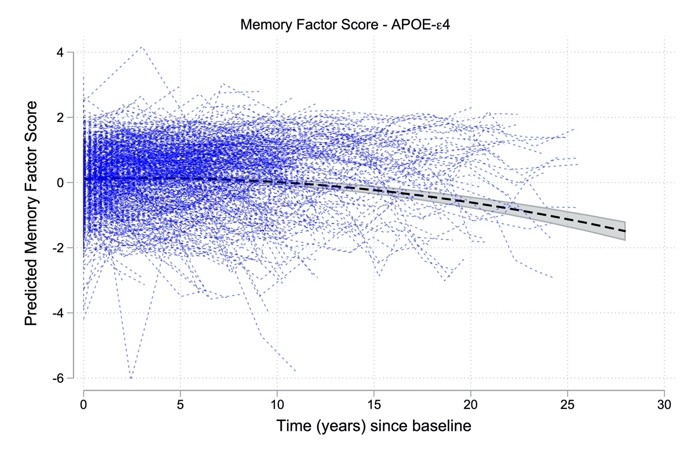

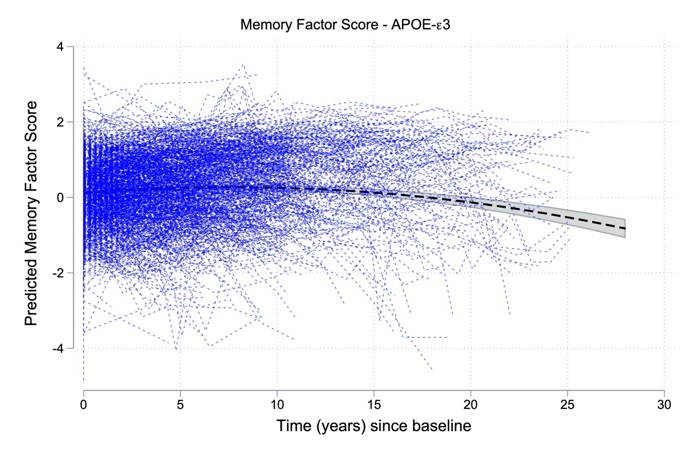

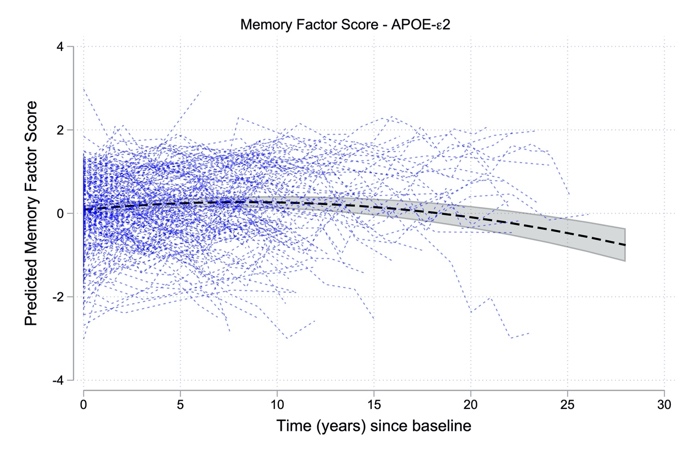

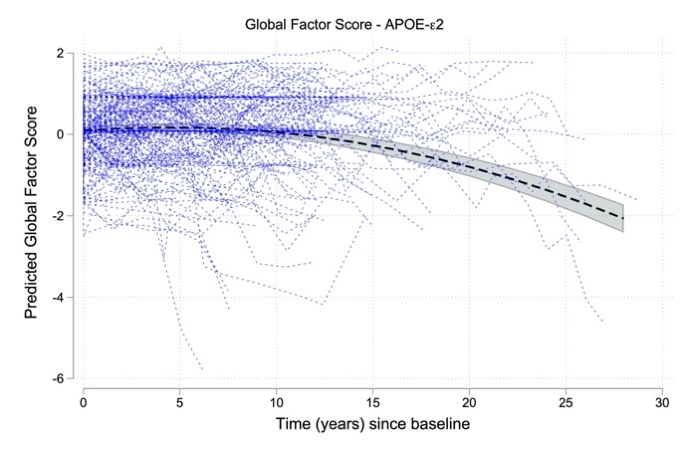


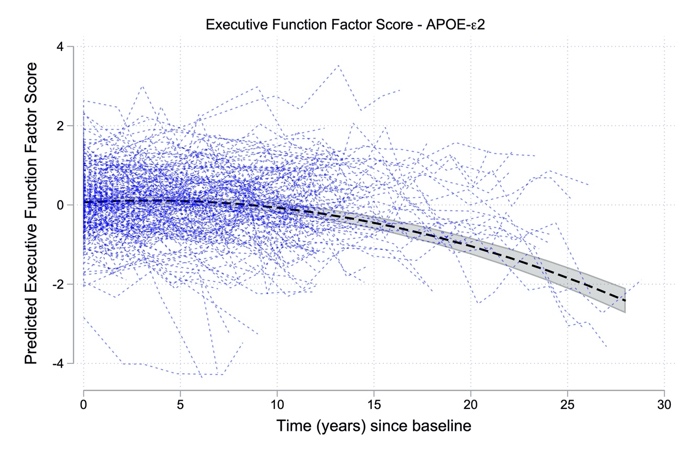

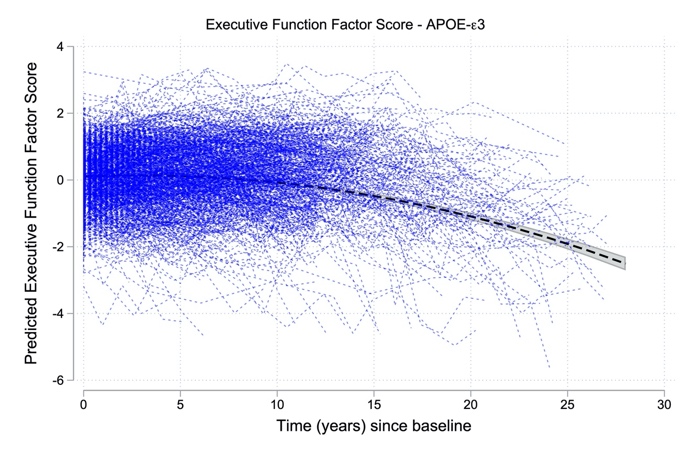

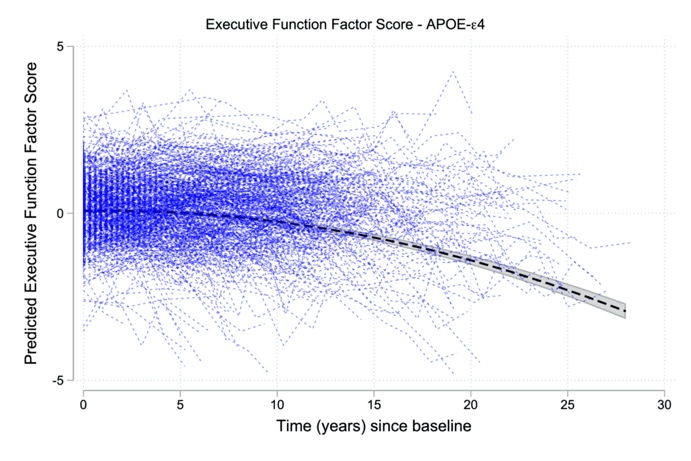


Supplementary Figure 1b. Spaghetti plots illustrating participant trajectories and estimated cognitive change for high (left) vs. low (right) AD-PRS_w/oAPOE_, by median split. Cognitive change is shown separately for the global (top row), episodic memory (middle row), and executive function (bottom row) factor scores based on the full models, as shown in Table 3. Sample means were used in the estimation of all other covariates. The AD-PRS_w/oAPOE_ x time interactions were significant for the global and executive function factor scores, but not for the memory factor score.


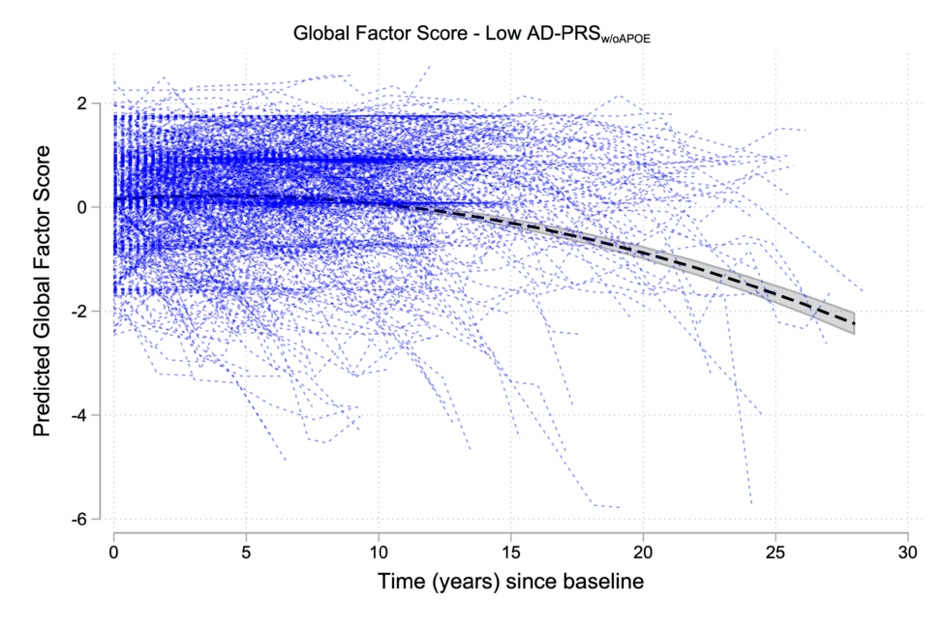

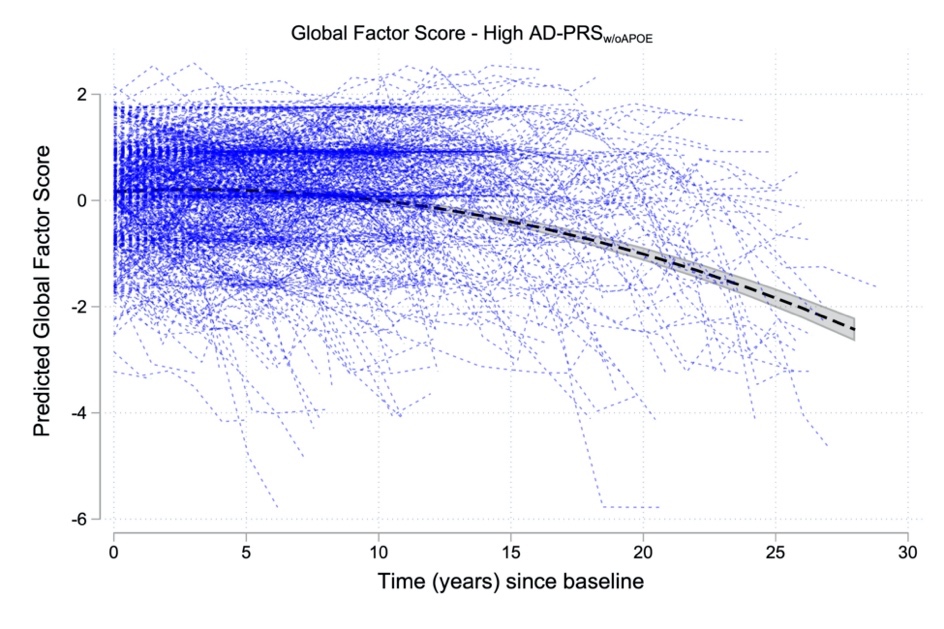

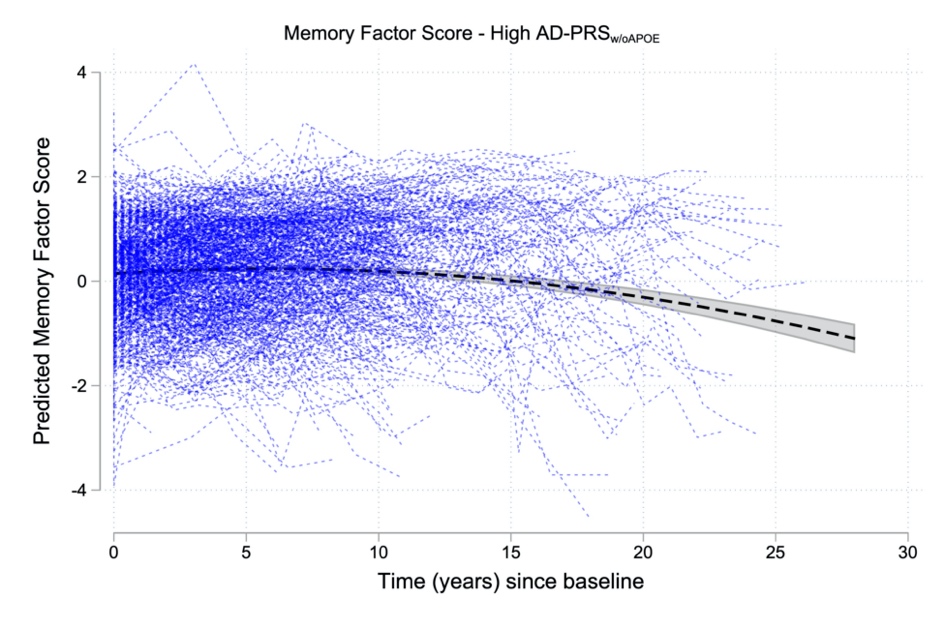

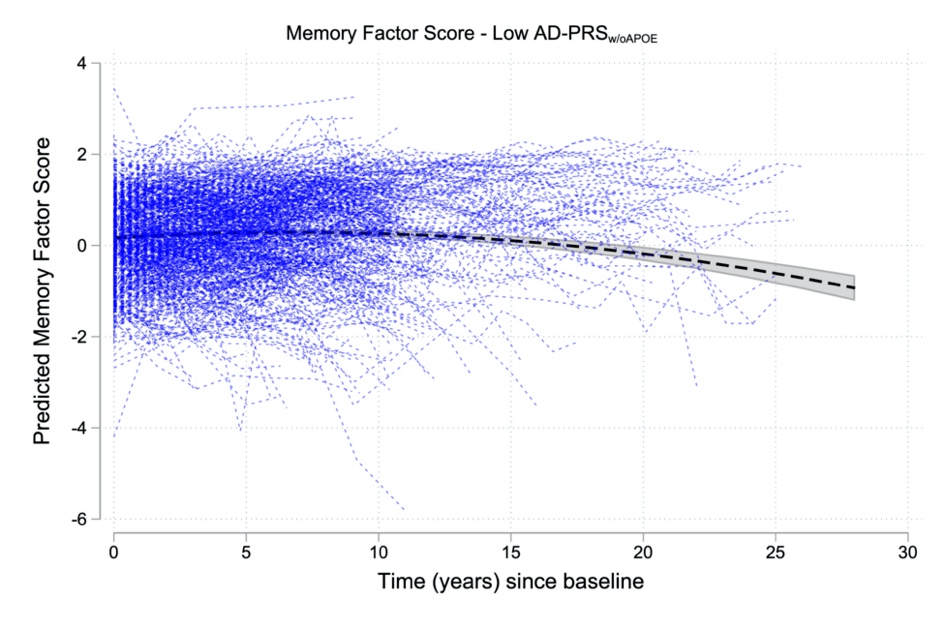

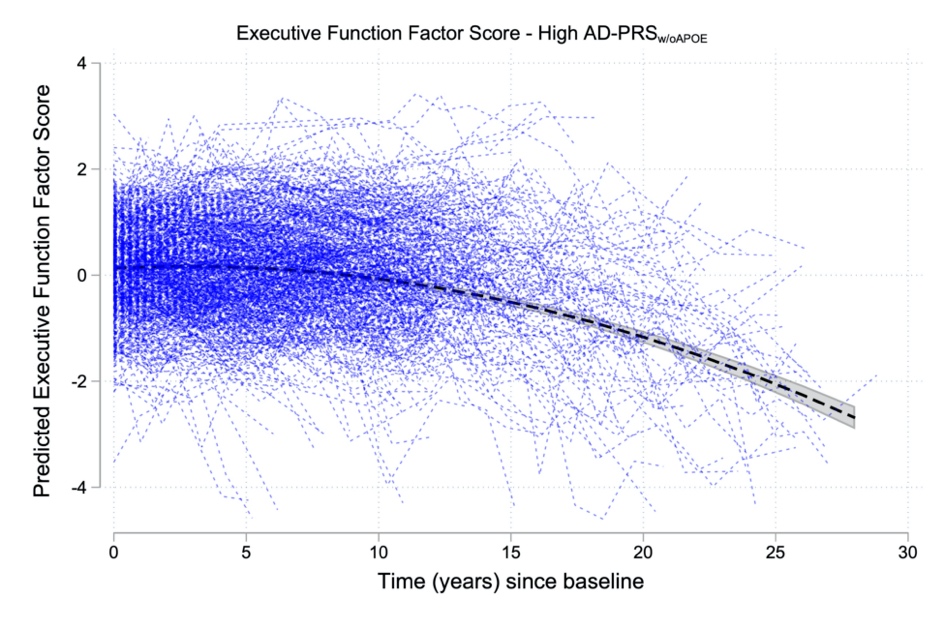

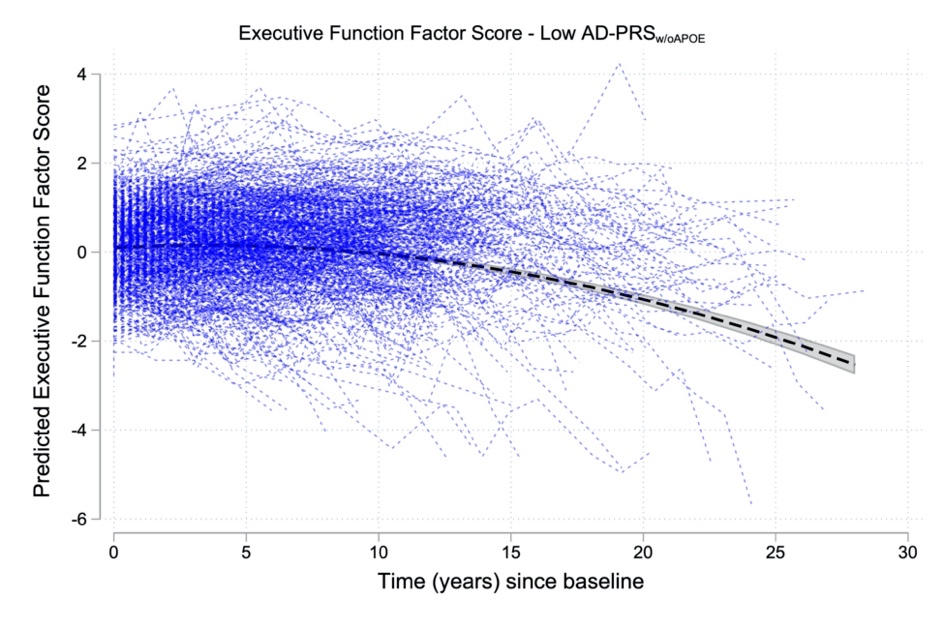

Supplement: Supplementary file 1 — Additional file 1: Supplementary Table 1. Cognitive tests included in the harmonized cognitive factor scores, by cohort. Supplementary Table 2. APOE genotyping approaches used by each PAC cohort. Supplementary Table 3. Baseline participant characteristics, by cohort, for APOE analyses. Values reflect mean (SD) unless otherwise indicated. Supplementary Table 4. Baseline participant characteristics, by cohort, for AD-PRS analyses. Values reflect mean (SD) unless otherwise indicated. Supplementary Table 5. Mixed-effects model results for AD-PRSAPOE and CR in relationship to cognitive trajectories. Supplementary Table 6. Descriptive statistics by follow-up diagnosis and mixed-effects model results for AD genetic risk and CR in relationship to cognitive trajectories, excluding individuals who progressed from normal cognition to MCI or dementia. Supplementary Table 7. Mixed-effects model results for AD genetic risk and the components of the CR index score in relationship to cognitive trajectories. Supplementary Table 8. Mixed-effects model results for AD-PRSw/oAPOE and CR in relationship to cognitive trajectories, a) excluding related individuals and b) covarying for population PCs. Supplementary Table 9. Mixed-effects model results for AD-PRSAPOE and CR in relationship to cognitive trajectories, a) excluding related individuals and b) covarying for population PCs. Supplementary Figure 1. Spaghetti plots illustrating participant trajectories and estimated cognitive change by AD genetic risk profiles. [file 13195_2023_1206_MOESM1_ESM.docx]
